# Supplementary figures and images for: Psychosocial hierarchies of modifiable risk for Alzheimer’s disease: A networks analysis
Source: PLoS One. 2026 Mar 6;21(3):e0333148. doi: 10.1371/journal.pone.0333148 (PMC12965608; doi:10.1371/journal.pone.0333148)

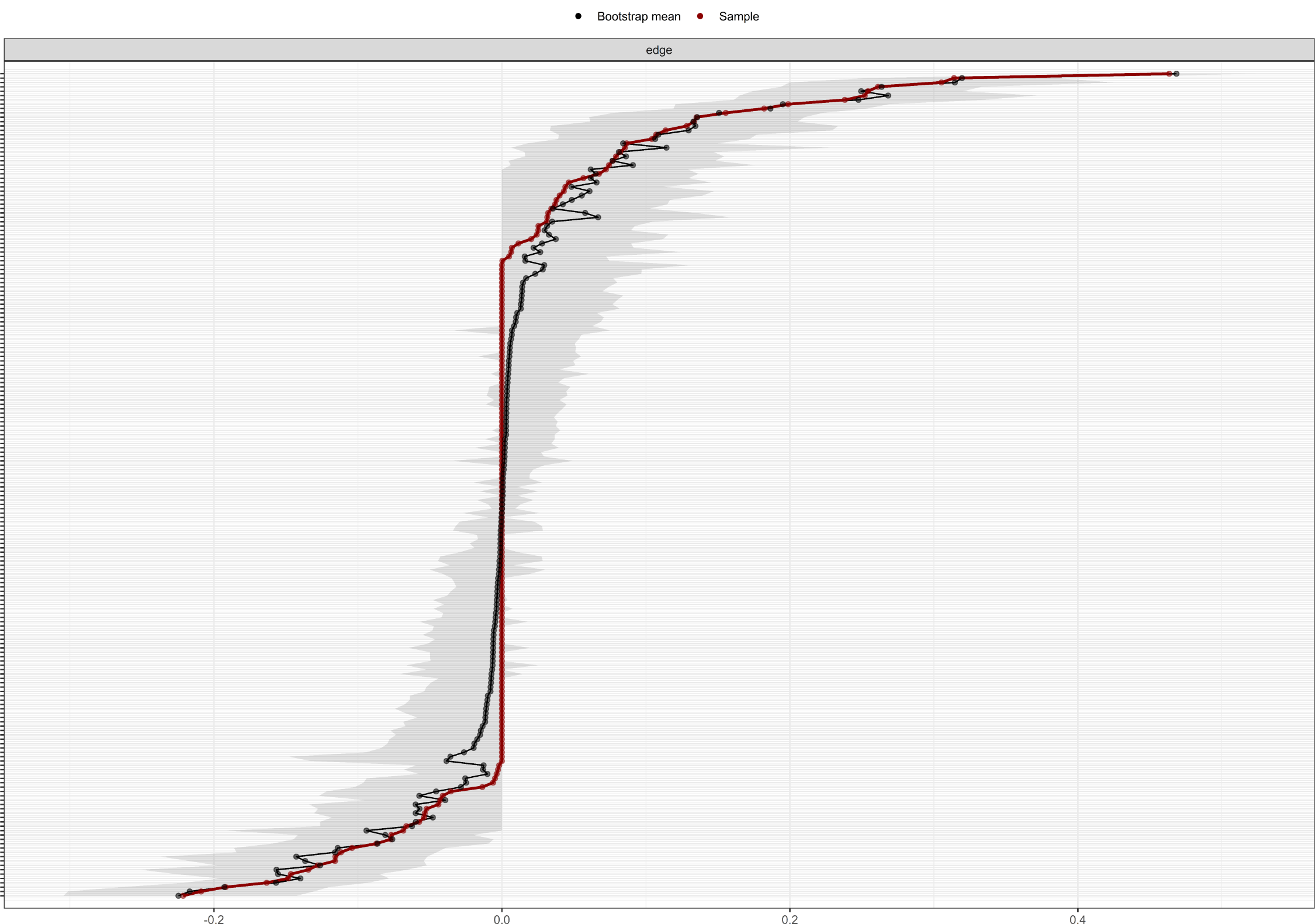

Supplement: S1 Fig — Plot contrasts estimated RPCN edge weights against bootstrapped mean and distribution range. Red dots indicate original sample edge weights; black dots represent bootstrapped means across 10,0000 resampled iterations. Grey shadow indicates the range of resamples edge weight. (TIF) [file pone.0333148.s001.tif]

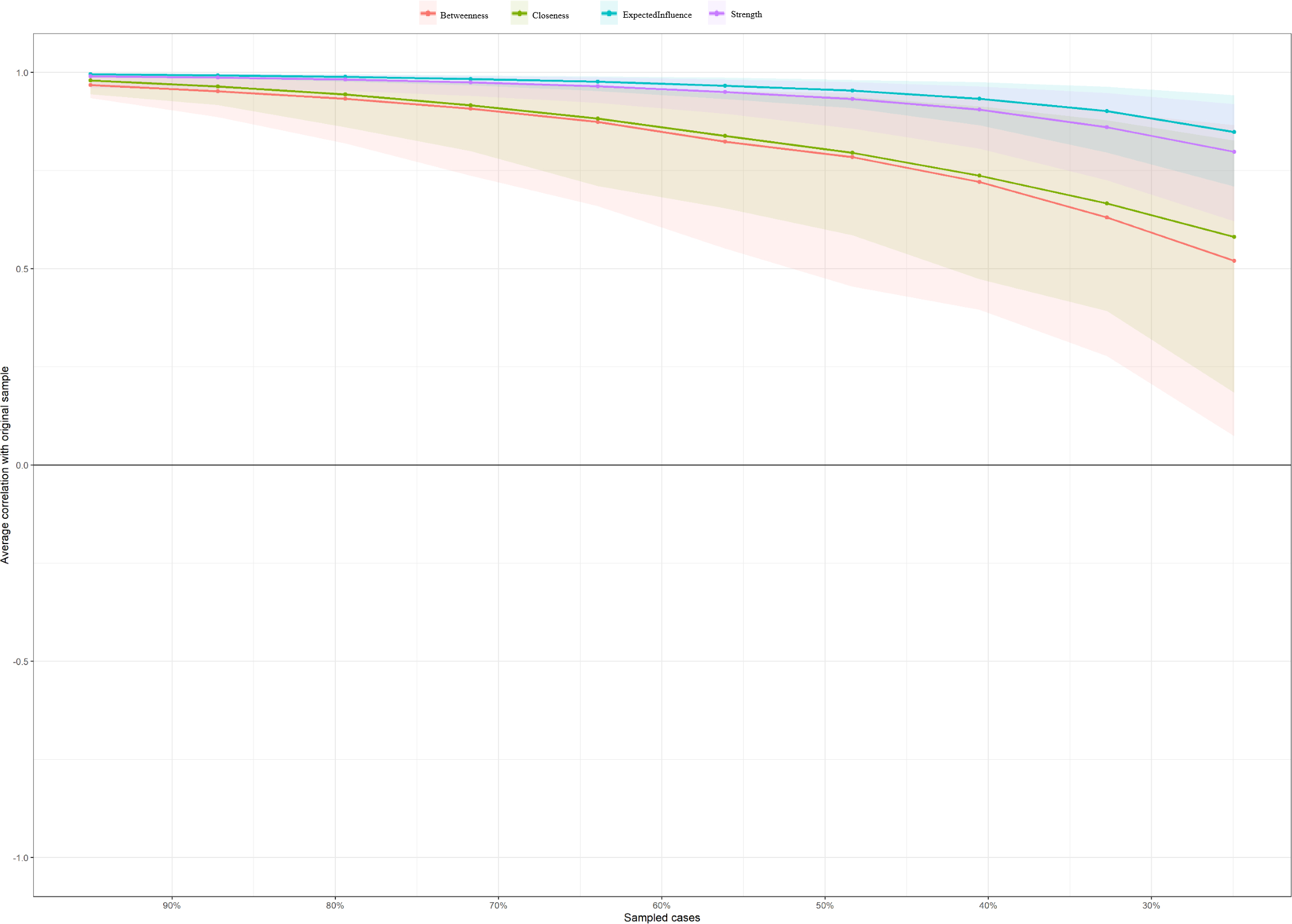

Supplement: S2 Fig — Plot depicts outcomes of case-dropping bootstraps assessing CS-coefficient stability. Colored opaque widths indicate error of estimation. (TIF) [file pone.0333148.s002.tif]
