# Supplementary material for: Psychosocial hierarchies of modifiable risk for Alzheimer’s disease: A networks analysis
Source: PLoS One. 2026 Mar 6;21(3):e0333148. doi: 10.1371/journal.pone.0333148 (PMC12965608; doi:10.1371/journal.pone.0333148)
Supplement: S1 Table — Depicted weight = non-robust glasso penalized values, displayed in the RPCN (Fig 2). * Attention to the management of that condition. † Robust weight estimates, across all bootstraps. ‡ Robust weight estimates, averaged across bootstraps where edge was present (reported). (DOCX) [file pone.0333148.s003.docx]

**S1 Table. Bootstrapped regularized partial correlation network edge weights and associated statistics.**

|  |  |  | Bootstrapped RPCN Coefficients | | | | | | | |
| --- | --- | --- | --- | --- | --- | --- | --- | --- | --- | --- |
|  |  |  | Overall^†^ | | | | Non-zero*^‡^* | | | |
| Edge | |  |  |  | 95% CI | |  |  | 95% CI | |
| Node A | Node B | Depicted Weight | Mean | SD | Lower | Upper | Mean | SD | Lower | Upper |
| Perceived stress | Chronic stress | 0.463 | 0.468 | 0.028 | 0.408 | 0.519 | 0.468 | 0.028 | 0.414 | 0.523 |
| Depression | Anxiety | 0.314 | 0.320 | 0.033 | 0.248 | 0.380 | 0.320 | 0.033 | 0.253 | 0.385 |
| Cholesterol^*^ | Diabetes^*^ | 0.305 | 0.315 | 0.058 | 0.190 | 0.420 | 0.315 | 0.058 | 0.199 | 0.425 |
| Perceived stress | Recent stressors | 0.261 | 0.264 | 0.035 | 0.191 | 0.331 | 0.264 | 0.035 | 0.194 | 0.333 |
| Perceived stress | Anxiety | 0.254 | 0.250 | 0.037 | 0.180 | 0.329 | 0.250 | 0.037 | 0.174 | 0.320 |
| Gender | Marital status | 0.252 | 0.268 | 0.052 | 0.148 | 0.356 | 0.268 | 0.052 | 0.165 | 0.371 |
| Alcohol | Gender | 0.238 | 0.248 | 0.044 | 0.151 | 0.325 | 0.248 | 0.044 | 0.161 | 0.334 |
| Chronic stress | Anxiety | 0.199 | 0.195 | 0.038 | 0.123 | 0.274 | 0.195 | 0.038 | 0.121 | 0.269 |
| Cognitive activity | Resilient coping | 0.182 | 0.187 | 0.034 | 0.114 | 0.251 | 0.187 | 0.034 | 0.119 | 0.253 |
| Chronic stress | Recent stressors | 0.155 | 0.151 | 0.037 | 0.082 | 0.229 | 0.151 | 0.037 | 0.077 | 0.223 |
| Physical activity | Social support | 0.135 | 0.136 | 0.038 | 0.059 | 0.211 | 0.136 | 0.038 | 0.061 | 0.210 |
| Cognitive activity | Social support | 0.133 | 0.133 | 0.037 | 0.060 | 0.207 | 0.133 | 0.037 | 0.061 | 0.205 |
| Cholesterol^*^ | Blood pressure^*^ | 0.128 | 0.134 | 0.051 | 0.027 | 0.230 | 0.135 | 0.050 | 0.038 | 0.233 |
| Age | Gender | 0.114 | 0.130 | 0.050 | 0.014 | 0.213 | 0.130 | 0.049 | 0.038 | 0.230 |
| Chronic stress | Depression | 0.107 | 0.109 | 0.035 | 0.036 | 0.178 | 0.109 | 0.035 | 0.040 | 0.177 |
| Alcohol | IRSAD decile | 0.104 | 0.106 | 0.034 | 0.036 | 0.172 | 0.107 | 0.034 | 0.039 | 0.172 |
| Depression | Recent stressors | 0.087 | 0.084 | 0.033 | 0.020 | 0.153 | 0.085 | 0.033 | 0.021 | 0.150 |
| Cholesterol^*^ | Gender | 0.085 | 0.114 | 0.055 | -0.024 | 0.195 | 0.117 | 0.053 | 0.022 | 0.229 |
| IRSAD decile | Social support | 0.082 | 0.081 | 0.033 | 0.016 | 0.149 | 0.082 | 0.032 | 0.020 | 0.147 |
| Physical activity | MIND diet | 0.079 | 0.086 | 0.035 | 0.010 | 0.149 | 0.087 | 0.034 | 0.022 | 0.154 |
| Social support | Resilient coping | 0.077 | 0.077 | 0.036 | 0.006 | 0.148 | 0.078 | 0.034 | 0.015 | 0.148 |
| MIND diet | Age | 0.074 | 0.091 | 0.042 | -0.009 | 0.158 | 0.093 | 0.040 | 0.021 | 0.176 |
| MIND diet | Social support | 0.072 | 0.062 | 0.034 | 0.005 | 0.139 | 0.065 | 0.031 | 0.009 | 0.130 |
| Cognitive activity | MIND diet | 0.067 | 0.065 | 0.036 | -0.005 | 0.140 | 0.068 | 0.034 | 0.008 | 0.137 |
| Physical activity | Alcohol | 0.057 | 0.062 | 0.034 | -0.011 | 0.124 | 0.065 | 0.032 | 0.010 | 0.130 |
| Age | Social support | 0.046 | 0.066 | 0.039 | -0.032 | 0.124 | 0.070 | 0.036 | 0.010 | 0.147 |
| BMI | Depression | 0.044 | 0.048 | 0.031 | -0.018 | 0.106 | 0.053 | 0.028 | 0.006 | 0.114 |
| MIND diet | Marital status | 0.043 | 0.061 | 0.042 | -0.040 | 0.126 | 0.069 | 0.037 | 0.009 | 0.151 |
| Marital status | Social support | 0.040 | 0.055 | 0.040 | -0.039 | 0.120 | 0.064 | 0.036 | 0.007 | 0.142 |
| Physical activity | Resilient coping | 0.038 | 0.049 | 0.033 | -0.028 | 0.103 | 0.055 | 0.030 | 0.007 | 0.119 |
| Physical activity | Blood pressure^*^ | 0.037 | 0.042 | 0.034 | -0.030 | 0.104 | 0.051 | 0.030 | 0.005 | 0.117 |
| Perceived stress | Depression | 0.034 | 0.035 | 0.030 | -0.027 | 0.095 | 0.044 | 0.028 | 0.003 | 0.106 |
| Physical activity | Education | 0.032 | 0.058 | 0.037 | -0.043 | 0.107 | 0.065 | 0.033 | 0.008 | 0.136 |
| Resilient coping | Recent stressors | 0.031 | 0.067 | 0.045 | -0.059 | 0.122 | 0.077 | 0.040 | 0.010 | 0.162 |
| Cholesterol^*^ | Resilient coping | 0.031 | 0.035 | 0.032 | -0.032 | 0.094 | 0.046 | 0.028 | 0.004 | 0.109 |
| MIND diet | IRSAD decile | 0.025 | 0.031 | 0.028 | -0.030 | 0.081 | 0.040 | 0.025 | 0.003 | 0.096 |
| BMI | Recent stressors | 0.025 | 0.030 | 0.027 | -0.028 | 0.079 | 0.039 | 0.024 | 0.003 | 0.093 |
| MIND diet | Diabetes^*^ | 0.024 | 0.033 | 0.036 | -0.047 | 0.096 | 0.049 | 0.033 | 0.003 | 0.125 |
| Alcohol | Social support | 0.020 | 0.037 | 0.034 | -0.047 | 0.088 | 0.050 | 0.030 | 0.005 | 0.116 |
| Cognitive activity | IRSAD decile | 0.011 | 0.028 | 0.027 | -0.043 | 0.066 | 0.039 | 0.025 | 0.003 | 0.095 |
| Cholesterol^*^ | Social support | 0.007 | 0.022 | 0.028 | -0.049 | 0.063 | 0.039 | 0.027 | 0.002 | 0.100 |
| Blood pressure^*^ | Diabetes^*^ | 0.006 | 0.027 | 0.037 | -0.068 | 0.081 | 0.048 | 0.039 | -0.014 | 0.136 |
| MIND diet | Resilient coping | 0.005 | 0.016 | 0.022 | -0.039 | 0.049 | 0.030 | 0.022 | 0.002 | 0.083 |
| Marital status | IRSAD decile | 0.000 | 0.016 | 0.023 | -0.045 | 0.045 | 0.032 | 0.022 | 0.002 | 0.084 |
| Cholesterol^*^ | Alcohol | - | -0.039 | 0.040 | -0.080 | 0.080 | 0.011 | 0.021 | -0.031 | 0.056 |
| Diabetes^*^ | Gender | - | -0.036 | 0.045 | -0.090 | 0.090 | -0.014 | 0.027 | -0.073 | 0.037 |
| Alcohol | Resilient coping | - | -0.026 | 0.029 | -0.058 | 0.058 | 0.018 | 0.026 | -0.039 | 0.075 |
| BMI | Age | - | -0.020 | 0.026 | -0.051 | 0.051 | -0.020 | 0.021 | -0.070 | 0.019 |
| MIND diet | Education | - | -0.019 | 0.024 | -0.049 | 0.049 | -0.042 | 0.027 | -0.101 | -0.003 |
| IRSAD decile | Resilient coping | - | -0.017 | 0.024 | -0.048 | 0.048 | -0.019 | 0.017 | -0.059 | 0.006 |
| BMI | Marital status | - | -0.015 | 0.022 | -0.044 | 0.044 | 0.054 | 0.040 | 0.001 | 0.147 |
| BMI | Anxiety | - | -0.015 | 0.023 | -0.046 | 0.046 | 0.026 | 0.024 | -0.016 | 0.080 |
| BMI | Social support | - | -0.013 | 0.020 | -0.040 | 0.040 | 0.022 | 0.021 | -0.007 | 0.079 |
| Cholesterol^*^ | Recent stressors | - | -0.012 | 0.020 | -0.040 | 0.040 | -0.022 | 0.018 | -0.066 | -0.001 |
| MIND diet | Chronic stress | - | -0.012 | 0.017 | -0.035 | 0.035 | -0.024 | 0.019 | -0.071 | -0.001 |
| Age | Recent stressors | - | -0.011 | 0.019 | -0.039 | 0.039 | 0.030 | 0.023 | 0.001 | 0.086 |
| Diabetes^*^ | Education | - | -0.011 | 0.022 | -0.043 | 0.043 | -0.023 | 0.018 | -0.067 | -0.001 |
| Physical activity | Recent stressors | - | -0.011 | 0.019 | -0.039 | 0.039 | 0.033 | 0.023 | 0.002 | 0.088 |
| Marital status | Resilient coping | - | -0.010 | 0.019 | -0.037 | 0.037 | 0.044 | 0.028 | 0.003 | 0.108 |
| Cholesterol^*^ | IRSAD decile | - | -0.010 | 0.019 | -0.037 | 0.037 | 0.030 | 0.021 | 0.002 | 0.080 |
| Social support | Anxiety | - | -0.008 | 0.015 | -0.031 | 0.031 | 0.020 | 0.020 | -0.019 | 0.065 |
| Marital status | Depression | - | -0.008 | 0.015 | -0.030 | 0.030 | -0.029 | 0.021 | -0.080 | -0.002 |
| IRSAD decile | Recent stressors | - | -0.008 | 0.014 | -0.029 | 0.029 | 0.025 | 0.022 | -0.009 | 0.078 |
| Chronic stress | IRSAD decile | - | -0.007 | 0.013 | -0.025 | 0.025 | 0.029 | 0.022 | 0.001 | 0.083 |
| Cognitive activity | Diabetes^*^ | - | -0.007 | 0.021 | -0.043 | 0.043 | -0.008 | 0.026 | -0.056 | 0.051 |
| Anxiety | Resilient coping | - | -0.007 | 0.015 | -0.029 | 0.029 | -0.022 | 0.032 | -0.091 | 0.046 |
| Diabetes^*^ | BMI | - | -0.006 | 0.021 | -0.042 | 0.042 | 0.016 | 0.025 | -0.037 | 0.067 |
| Alcohol | Recent stressors | - | -0.006 | 0.014 | -0.028 | 0.028 | 0.021 | 0.024 | -0.032 | 0.075 |
| Blood pressure^*^ | Perceived stress | - | -0.006 | 0.014 | -0.029 | 0.029 | -0.007 | 0.032 | -0.072 | 0.050 |
| Education | Recent stressors | - | -0.006 | 0.014 | -0.028 | 0.028 | -0.023 | 0.020 | -0.072 | -0.001 |
| Blood pressure^*^ | Gender | - | -0.006 | 0.020 | -0.039 | 0.039 | -0.024 | 0.024 | -0.079 | 0.024 |
| Physical activity | Chronic stress | - | -0.006 | 0.014 | -0.029 | 0.029 | 0.021 | 0.020 | -0.012 | 0.070 |
| Cognitive activity | Perceived stress | - | -0.006 | 0.013 | -0.026 | 0.026 | -0.035 | 0.024 | -0.089 | -0.002 |
| Blood pressure^*^ | Recent stressors | - | -0.006 | 0.016 | -0.031 | 0.031 | -0.036 | 0.025 | -0.094 | -0.003 |
| Age | Anxiety | - | -0.006 | 0.013 | -0.027 | 0.027 | 0.017 | 0.023 | -0.028 | 0.066 |
| Alcohol | BMI | - | -0.005 | 0.014 | -0.027 | 0.027 | -0.011 | 0.029 | -0.068 | 0.052 |
| Diabetes^*^ | IRSAD decile | - | -0.005 | 0.017 | -0.034 | 0.034 | 0.024 | 0.021 | -0.008 | 0.073 |
| Cholesterol^*^ | Anxiety | - | -0.004 | 0.012 | -0.023 | 0.023 | -0.028 | 0.021 | -0.076 | -0.001 |
| Social support | Recent stressors | - | -0.004 | 0.011 | -0.022 | 0.022 | -0.031 | 0.022 | -0.083 | -0.002 |
| Gender | Resilient coping | - | -0.004 | 0.014 | -0.029 | 0.029 | 0.009 | 0.018 | -0.031 | 0.048 |
| Education | Social support | - | -0.004 | 0.011 | -0.023 | 0.023 | 0.020 | 0.016 | 0.001 | 0.058 |
| Cholesterol^*^ | MIND diet | - | -0.004 | 0.014 | -0.027 | 0.027 | -0.057 | 0.036 | -0.141 | -0.005 |
| Diabetes^*^ | Perceived stress | - | -0.003 | 0.012 | -0.024 | 0.024 | -0.020 | 0.018 | -0.062 | 0.004 |
| Alcohol | Depression | - | -0.003 | 0.010 | -0.020 | 0.020 | 0.017 | 0.025 | -0.036 | 0.070 |
| Chronic stress | Education | - | -0.003 | 0.009 | -0.017 | 0.017 | 0.037 | 0.027 | 0.001 | 0.101 |
| MIND diet | Perceived stress | - | -0.003 | 0.009 | -0.018 | 0.018 | 0.005 | 0.029 | -0.054 | 0.066 |
| Education | Depression | - | -0.003 | 0.010 | -0.019 | 0.019 | -0.021 | 0.027 | -0.078 | 0.035 |
| BMI | Gender | - | -0.002 | 0.013 | -0.027 | 0.027 | -0.029 | 0.022 | -0.082 | -0.001 |
| Cholesterol^*^ | Marital status | - | -0.002 | 0.016 | -0.033 | 0.033 | 0.012 | 0.029 | -0.047 | 0.072 |
| Alcohol | Age | - | -0.002 | 0.011 | -0.021 | 0.021 | -0.010 | 0.034 | -0.080 | 0.056 |
| Gender | Education | - | -0.002 | 0.015 | -0.031 | 0.031 | 0.015 | 0.029 | -0.030 | 0.075 |
| Blood pressure^*^ | Marital status | - | -0.001 | 0.014 | -0.028 | 0.028 | -0.029 | 0.023 | -0.083 | -0.001 |
| Blood pressure^*^ | Depression | - | -0.001 | 0.010 | -0.020 | 0.020 | 0.020 | 0.024 | -0.030 | 0.070 |
| IRSAD decile | Depression | - | -0.001 | 0.006 | -0.012 | 0.012 | 0.024 | 0.019 | 0.000 | 0.068 |
| Physical activity | Age | - | -0.001 | 0.008 | -0.015 | 0.015 | 0.016 | 0.023 | -0.038 | 0.062 |
| Perceived stress | IRSAD decile | - | -0.001 | 0.005 | -0.010 | 0.010 | 0.027 | 0.020 | 0.001 | 0.076 |
| Perceived stress | Education | - | -0.001 | 0.005 | -0.011 | 0.011 | -0.027 | 0.034 | -0.102 | 0.040 |
| Diabetes^*^ | Depression | - | -0.001 | 0.013 | -0.026 | 0.026 | 0.005 | 0.028 | -0.054 | 0.063 |
| Diabetes^*^ | Recent stressors | - | -0.001 | 0.012 | -0.025 | 0.025 | -0.022 | 0.017 | -0.064 | -0.001 |
| Physical activity | Marital status | - | 0.000 | 0.011 | -0.022 | 0.022 | 0.015 | 0.021 | -0.029 | 0.062 |
| Perceived stress | Social support | - | 0.000 | 0.005 | -0.010 | 0.010 | 0.014 | 0.018 | -0.018 | 0.054 |
| Diabetes^*^ | Anxiety | - | 0.000 | 0.011 | -0.023 | 0.023 | -0.001 | 0.027 | -0.055 | 0.055 |
| Chronic stress | Marital status | - | 0.000 | 0.006 | -0.013 | 0.013 | 0.007 | 0.034 | -0.061 | 0.077 |
| Education | Anxiety | - | 0.000 | 0.006 | -0.013 | 0.013 | -0.022 | 0.034 | -0.091 | 0.053 |
| Gender | Anxiety | - | 0.000 | 0.009 | -0.018 | 0.018 | 0.029 | 0.027 | -0.023 | 0.090 |
| BMI | Perceived stress | - | 0.001 | 0.005 | -0.010 | 0.010 | -0.005 | 0.030 | -0.063 | 0.062 |
| Cognitive activity | Marital status | - | 0.001 | 0.010 | -0.019 | 0.019 | -0.061 | 0.044 | -0.163 | -0.002 |
| Cognitive activity | Chronic stress | - | 0.001 | 0.005 | -0.009 | 0.009 | -0.019 | 0.031 | -0.080 | 0.045 |
| Cholesterol^*^ | Cognitive activity | - | 0.001 | 0.011 | -0.022 | 0.022 | -0.030 | 0.027 | -0.090 | 0.019 |
| Marital status | Anxiety | - | 0.001 | 0.007 | -0.013 | 0.013 | 0.032 | 0.027 | -0.009 | 0.095 |
| Alcohol | Anxiety | - | 0.001 | 0.007 | -0.014 | 0.014 | 0.021 | 0.038 | -0.057 | 0.102 |
| IRSAD decile | Anxiety | - | 0.001 | 0.006 | -0.012 | 0.012 | -0.016 | 0.022 | -0.067 | 0.026 |
| Cholesterol^*^ | Perceived stress | - | 0.001 | 0.010 | -0.020 | 0.020 | -0.003 | 0.028 | -0.059 | 0.056 |
| Diabetes^*^ | Age | - | 0.002 | 0.017 | -0.034 | 0.034 | 0.013 | 0.027 | -0.039 | 0.074 |
| Cognitive activity | Recent stressors | - | 0.002 | 0.009 | -0.018 | 0.018 | 0.024 | 0.021 | -0.009 | 0.071 |
| Alcohol | Perceived stress | - | 0.002 | 0.009 | -0.017 | 0.017 | 0.024 | 0.019 | 0.000 | 0.071 |
| Cholesterol^*^ | Education | - | 0.002 | 0.013 | -0.026 | 0.026 | 0.033 | 0.026 | 0.001 | 0.094 |
| BMI | Resilient coping | - | 0.002 | 0.010 | -0.020 | 0.020 | -0.034 | 0.023 | -0.087 | -0.003 |
| Diabetes^*^ | Chronic stress | - | 0.002 | 0.012 | -0.024 | 0.024 | -0.018 | 0.015 | -0.055 | -0.001 |
| Cognitive activity | Anxiety | - | 0.003 | 0.010 | -0.020 | 0.020 | 0.028 | 0.023 | -0.008 | 0.081 |
| Blood pressure^*^ | IRSAD decile | - | 0.003 | 0.012 | -0.024 | 0.024 | -0.025 | 0.018 | -0.069 | -0.001 |
| Education | Resilient coping | - | 0.003 | 0.010 | -0.021 | 0.021 | 0.003 | 0.027 | -0.052 | 0.060 |
| MIND diet | Anxiety | - | 0.003 | 0.010 | -0.021 | 0.021 | -0.018 | 0.027 | -0.074 | 0.042 |
| Blood pressure^*^ | Chronic stress | - | 0.003 | 0.010 | -0.021 | 0.021 | 0.032 | 0.025 | 0.001 | 0.089 |
| Perceived stress | Marital status | - | 0.003 | 0.011 | -0.022 | 0.022 | 0.017 | 0.027 | -0.044 | 0.070 |
| Cognitive activity | Alcohol | - | 0.003 | 0.012 | -0.024 | 0.024 | -0.009 | 0.034 | -0.083 | 0.057 |
| Gender | IRSAD decile | - | 0.003 | 0.013 | -0.027 | 0.027 | 0.025 | 0.034 | -0.038 | 0.099 |
| Cognitive activity | Blood pressure^*^ | - | 0.003 | 0.012 | -0.025 | 0.025 | 0.014 | 0.016 | -0.017 | 0.049 |
| Physical activity | Cholesterol^*^ | - | 0.004 | 0.013 | -0.027 | 0.027 | -0.035 | 0.023 | -0.089 | -0.003 |
| Cholesterol^*^ | Age | - | 0.004 | 0.013 | -0.027 | 0.027 | -0.012 | 0.016 | -0.047 | 0.026 |
| Alcohol | Blood pressure^*^ | - | 0.004 | 0.014 | -0.028 | 0.028 | -0.022 | 0.017 | -0.063 | -0.001 |
| Physical activity | IRSAD decile | - | 0.004 | 0.011 | -0.022 | 0.022 | 0.002 | 0.021 | -0.042 | 0.045 |
| Age | Education | - | 0.004 | 0.012 | -0.024 | 0.024 | 0.019 | 0.020 | -0.021 | 0.064 |
| Gender | Recent stressors | - | 0.004 | 0.017 | -0.034 | 0.034 | -0.019 | 0.019 | -0.061 | 0.016 |
| BMI | Chronic stress | - | 0.005 | 0.011 | -0.022 | 0.022 | -0.019 | 0.020 | -0.066 | 0.020 |
| MIND diet | Recent stressors | - | 0.005 | 0.014 | -0.028 | 0.028 | -0.023 | 0.019 | -0.068 | 0.000 |
| Blood pressure^*^ | Social support | - | 0.005 | 0.014 | -0.029 | 0.029 | -0.024 | 0.018 | -0.069 | -0.001 |
| Physical activity | Diabetes^*^ | - | 0.005 | 0.017 | -0.034 | 0.034 | -0.020 | 0.017 | -0.061 | 0.000 |
| MIND diet | Alcohol | - | 0.005 | 0.014 | -0.028 | 0.028 | 0.008 | 0.021 | -0.033 | 0.050 |
| BMI | Education | - | 0.006 | 0.014 | -0.028 | 0.028 | -0.029 | 0.021 | -0.079 | -0.001 |
| Physical activity | Cognitive activity | - | 0.006 | 0.014 | -0.028 | 0.028 | -0.023 | 0.018 | -0.066 | -0.001 |
| Blood pressure^*^ | Anxiety | - | 0.006 | 0.016 | -0.031 | 0.031 | 0.043 | 0.026 | 0.003 | 0.103 |
| Cognitive activity | BMI | - | 0.007 | 0.016 | -0.031 | 0.031 | -0.012 | 0.023 | -0.065 | 0.034 |
| Diabetes^*^ | Marital status | - | 0.007 | 0.024 | -0.048 | 0.048 | 0.016 | 0.025 | -0.038 | 0.067 |
| Alcohol | Marital status | - | 0.008 | 0.019 | -0.037 | 0.037 | 0.024 | 0.020 | 0.000 | 0.071 |
| Gender | Depression | - | 0.010 | 0.020 | -0.040 | 0.040 | 0.020 | 0.029 | -0.043 | 0.077 |
| Diabetes^*^ | Resilient coping | - | 0.010 | 0.021 | -0.042 | 0.042 | 0.037 | 0.029 | 0.000 | 0.107 |
| Blood pressure^*^ | Resilient coping | - | 0.011 | 0.019 | -0.039 | 0.039 | 0.020 | 0.017 | -0.001 | 0.061 |
| Cholesterol^*^ | BMI | - | 0.013 | 0.024 | -0.048 | 0.048 | -0.003 | 0.028 | -0.063 | 0.052 |
| Physical activity | Perceived stress | - | 0.013 | 0.022 | -0.045 | 0.045 | 0.033 | 0.024 | 0.002 | 0.091 |
| Diabetes^*^ | Social support | - | 0.014 | 0.024 | -0.047 | 0.047 | -0.029 | 0.022 | -0.086 | -0.002 |
| Physical activity | Gender | - | 0.014 | 0.025 | -0.050 | 0.050 | -0.024 | 0.020 | -0.075 | -0.001 |
| Age | IRSAD decile | - | 0.014 | 0.021 | -0.042 | 0.042 | -0.012 | 0.013 | -0.041 | 0.012 |
| Anxiety | Recent stressors | - | 0.014 | 0.022 | -0.044 | 0.044 | -0.010 | 0.015 | -0.043 | 0.023 |
| MIND diet | Blood pressure^*^ | - | 0.015 | 0.024 | -0.047 | 0.047 | -0.006 | 0.021 | -0.046 | 0.047 |
| Age | Resilient coping | - | 0.017 | 0.023 | -0.047 | 0.047 | 0.023 | 0.020 | -0.005 | 0.074 |
| Age | Depression | - | 0.023 | 0.030 | -0.060 | 0.060 | -0.019 | 0.014 | -0.052 | -0.001 |
| Physical activity | Anxiety | - | 0.028 | 0.029 | -0.059 | 0.059 | -0.017 | 0.013 | -0.050 | 0.000 |
| Alcohol | Diabetes^*^ | - | 0.029 | 0.040 | -0.080 | 0.080 | 0.002 | 0.024 | -0.043 | 0.049 |
| Alcohol | Chronic stress | -0.002 | -0.013 | 0.018 | -0.038 | 0.034 | -0.024 | 0.018 | -0.070 | -0.001 |
| Perceived stress | Age | -0.003 | -0.013 | 0.020 | -0.043 | 0.038 | -0.028 | 0.021 | -0.080 | -0.001 |
| Cholesterol^*^ | Chronic stress | -0.004 | -0.010 | 0.017 | -0.038 | 0.030 | -0.024 | 0.019 | -0.069 | -0.001 |
| Alcohol | Education | -0.005 | -0.025 | 0.029 | -0.062 | 0.052 | -0.040 | 0.026 | -0.101 | -0.003 |
| Blood pressure^*^ | Education | -0.006 | -0.025 | 0.029 | -0.065 | 0.052 | -0.041 | 0.027 | -0.104 | -0.003 |
| Perceived stress | Gender | -0.014 | -0.029 | 0.031 | -0.075 | 0.048 | -0.042 | 0.029 | -0.109 | -0.003 |
| Marital status | Education | -0.035 | -0.046 | 0.036 | -0.107 | 0.036 | -0.056 | 0.032 | -0.125 | -0.006 |
| Physical activity | Depression | -0.041 | -0.057 | 0.034 | -0.109 | 0.027 | -0.061 | 0.032 | -0.127 | -0.008 |
| Chronic stress | Social support | -0.043 | -0.039 | 0.026 | -0.096 | 0.010 | -0.043 | 0.024 | -0.097 | -0.004 |
| Cognitive activity | Age | -0.044 | -0.060 | 0.037 | -0.117 | 0.029 | -0.065 | 0.033 | -0.136 | -0.009 |
| Chronic stress | Resilient coping | -0.052 | -0.057 | 0.034 | -0.121 | 0.016 | -0.061 | 0.032 | -0.129 | -0.008 |
| Marital status | Recent stressors | -0.054 | -0.060 | 0.034 | -0.122 | 0.015 | -0.063 | 0.032 | -0.131 | -0.009 |
| Cholesterol^*^ | Depression | -0.054 | -0.048 | 0.034 | -0.122 | 0.013 | -0.054 | 0.031 | -0.120 | -0.006 |
| BMI | IRSAD decile | -0.057 | -0.060 | 0.033 | -0.124 | 0.009 | -0.063 | 0.031 | -0.127 | -0.009 |
| MIND diet | Depression | -0.066 | -0.062 | 0.032 | -0.130 | -0.003 | -0.064 | 0.030 | -0.126 | -0.010 |
| Gender | Social support | -0.068 | -0.094 | 0.049 | -0.167 | 0.030 | -0.098 | 0.046 | -0.192 | -0.017 |
| Cognitive activity | Depression | -0.077 | -0.081 | 0.031 | -0.139 | -0.015 | -0.082 | 0.030 | -0.142 | -0.023 |
| Depression | Resilient coping | -0.077 | -0.076 | 0.035 | -0.147 | -0.007 | -0.077 | 0.034 | -0.145 | -0.014 |
| Chronic stress | Gender | -0.087 | -0.086 | 0.037 | -0.162 | -0.012 | -0.088 | 0.036 | -0.160 | -0.018 |
| Perceived stress | Resilient coping | -0.104 | -0.114 | 0.037 | -0.179 | -0.029 | -0.114 | 0.037 | -0.186 | -0.041 |
| IRSAD decile | Education | -0.112 | -0.116 | 0.035 | -0.182 | -0.041 | -0.116 | 0.035 | -0.184 | -0.046 |
| Age | Marital status | -0.116 | -0.143 | 0.048 | -0.211 | -0.020 | -0.143 | 0.047 | -0.237 | -0.054 |
| Blood pressure^*^ | Age | -0.116 | -0.137 | 0.042 | -0.200 | -0.031 | -0.137 | 0.042 | -0.217 | -0.054 |
| MIND diet | BMI | -0.128 | -0.126 | 0.037 | -0.202 | -0.054 | -0.126 | 0.037 | -0.198 | -0.053 |
| MIND diet | Gender | -0.134 | -0.157 | 0.048 | -0.229 | -0.039 | -0.157 | 0.047 | -0.251 | -0.066 |
| Cognitive activity | Education | -0.147 | -0.155 | 0.036 | -0.218 | -0.075 | -0.155 | 0.036 | -0.226 | -0.085 |
| Chronic stress | Age | -0.148 | -0.140 | 0.030 | -0.209 | -0.088 | -0.140 | 0.030 | -0.196 | -0.078 |
| Social support | Depression | -0.163 | -0.157 | 0.033 | -0.229 | -0.098 | -0.157 | 0.033 | -0.220 | -0.093 |
| Physical activity | BMI | -0.192 | -0.193 | 0.037 | -0.266 | -0.118 | -0.193 | 0.037 | -0.264 | -0.120 |
| Blood pressure^*^ | BMI | -0.209 | -0.217 | 0.043 | -0.296 | -0.122 | -0.217 | 0.043 | -0.301 | -0.131 |
| Cognitive activity | Gender | -0.221 | -0.225 | 0.041 | -0.304 | -0.139 | -0.225 | 0.041 | -0.305 | -0.143 |

*Depicted weight = non-robust glasso penalized values, displayed in the RPCN (Figure 1). ^*^ Attention to the management of that condition. ^†^ Robust weight estimates, across all bootstraps.* *^‡^ Robust weight estimates, averaged across bootstraps where edge was present (reported).*
