# Supplementary material for: Psychosocial hierarchies of modifiable risk for Alzheimer’s disease: A networks analysis
Source: PLoS One. 2026 Mar 6;21(3):e0333148. doi: 10.1371/journal.pone.0333148 (PMC12965608; doi:10.1371/journal.pone.0333148)
Supplement: S2 Table — *Attention to the management of that condition. Table indicates single-node statistics, as they relate to variables contained in the RPCN (Fig 2), and accompanying scaled statistics (Z-scores). CS-coefficient descriptions detailed in methods. Variables are arranged in descending order of predictability. (DOCX) [file pone.0333148.s004.docx]

**S2 Table. Complete per-node regularized partial correlation network CS-coefficients.**

| Node |  | CS-coefficient (Raw) | | | |  | CS-coefficient (Z-Scored) | | | |
| --- | --- | --- | --- | --- | --- | --- | --- | --- | --- | --- |
|  | Predictability (R2) | Strength | ExpectedInfluence | Betweenness | Closeness |  | Strength | ExpectedInfluence | Betweenness | Closeness |
| Chronic stress | 0.59 | 1.26 | 0.59 | 25 | 0.003 |  | 1.95 | 1.11 | 0.78 | 0.48 |
| Perceived stress | 0.58 | 1.13 | 0.89 | 21 | 0.003 |  | 1.50 | 1.97 | 0.50 | 0.24 |
| Anxiety | 0.47 | 0.77 | 0.77 | 12 | 0.003 |  | -1.01 | 0.61 | -0.47 | 0.32 |
| Depression | 0.43 | 1.07 | 0.11 | 17 | 0.003 |  | 1.26 | -0.25 | 0.22 | 0.48 |
| Recent stressors | 0.36 | 0.61 | 0.51 | 0 | 0.003 |  | -0.35 | 0.88 | -0.96 | -1.13 |
| Social support | 0.24 | 0.89 | 0.34 | 18 | 0.004 |  | 0.63 | 0.41 | 0.29 | 0.83 |
| Cognitive activity | 0.23 | 0.88 | -0.09 | 37 | 0.004 |  | 0.61 | -0.82 | 1.62 | 1.46 |
| Resilient coping | 0.22 | 0.60 | 0.13 | 4 | 0.003 |  | -0.40 | -0.18 | -0.68 | -0.03 |
| Gender | 0.21 | 1.21 | 0.16 | 57 | 0.004 |  | 1.79 | -0.09 | 3.01 | 2.32 |
| Age | 0.19 | 0.66 | -0.19 | 22 | 0.004 |  | -0.18 | -1.09 | 0.57 | 0.92 |
| MIND diet | 0.17 | 0.72 | 0.06 | 5 | 0.003 |  | 0.03 | -0.37 | -0.61 | 0.13 |
| Physical activity | 0.16 | 0.61 | 0.14 | 5 | 0.003 |  | -0.36 | -0.14 | -0.61 | -0.47 |
| BMI | 0.16 | 0.66 | -0.52 | 8 | 0.003 |  | -0.20 | -2.01 | -0.40 | -0.42 |
| Cholesterol^*^ | 0.15 | 0.62 | 0.50 | 18 | 0.003 |  | -0.34 | 0.86 | 0.29 | -0.89 |
| Marital status | 0.11 | 0.54 | 0.13 | 0 | 0.003 |  | -0.61 | -0.18 | -0.96 | 0.34 |
| Diabetes^*^ | 0.10 | 0.34 | 0.34 | 0 | 0.002 |  | -1.33 | 0.40 | -0.96 | -1.81 |
| Alcohol | 0.10 | 0.43 | 0.41 | 7 | 0.003 |  | 0.20 | 1.61 | -0.13 | 0.22 |
| IRSAD decile | 0.10 | 0.39 | 0.05 | 1 | 0.003 |  | -1.13 | -0.40 | -0.89 | -1.36 |
| Blood pressure^*^ | 0.09 | 0.50 | -0.16 | 17 | 0.003 |  | -0.74 | -1.00 | 0.22 | -0.47 |
| Education | 0.08 | 0.34 | -0.27 | 2 | 0.003 |  | -1.33 | -1.32 | -0.82 | -1.15 |

*^*^ Attention to the management of that condition. Table indicates single-node statistics, as they relate to variables contained in the RPCN (Figure 1), and accompanying scaled statistics (Z-scores). CS-coefficient descriptions detailed in methods. Variables are arranged in descending order of predictability.*
