# Supplementary material for: Psychosocial hierarchies of modifiable risk for Alzheimer’s disease: A networks analysis
Source: PLoS One. 2026 Mar 6;21(3):e0333148. doi: 10.1371/journal.pone.0333148 (PMC12965608; doi:10.1371/journal.pone.0333148)
Supplement: S3 Table — * Arc coefficients for the given network. †Arc coefficients averaged across subnetworks where they occurred. ‡Arc coefficients averaged across all potential subnetworks. §Attention to the management of that condition. INT = Inverse normal transformation, NPN = Non-paranormal transformation, Disc5 = Five-level discretization transformation, Disc3 = Three-level discretization transformation, TABU = TABU list algorithm, HC = Hill-climbing algorithm, MMHC = max-min hill-climbing algorithm, H2PC = Hybrid HPC algorithm. BMI = Body mass index, MIND = Mediterranean-DASH Intervention for Neurodegenerative Delay, IRSAD = Index of Relative Socio-Economic Advantage and Disadvantage. (DOCX) [file pone.0333148.s005.docx]

**S3 Table. Complete Bayesian subnetwork arc statistics.**

| Arcs | |  |  | Observed^*^ | | Learned*^†^* | | Grand*^‡^* | |  |  |
| --- | --- | --- | --- | --- | --- | --- | --- | --- | --- | --- | --- |
| From | To | Algorithm | Transformation | Strength | Direction | Strength | Direction | Strength | Direction | Arc subnetwork presence | Arc retained |
| Physical activity | MIND diet | H2PC | Disc3 | 0.864 | 0.718 | 0.899 | 0.704 | 0.899 | 0.704 | 16 | Yes |
| Physical activity | BMI | H2PC | Disc3 | 0.999 | 0.582 | 0.973 | 0.563 | 0.912 | 0.528 | 15 | Yes |
| Cholesterol^§^ | Diabetes^§^ | H2PC | Disc3 | 1 | 0.553 | 1 | 0.534 | 0.750 | 0.400 | 12 | Yes |
| Cognitive activity | MIND diet | H2PC | Disc3 | 0.867 | 0.961 | 0.887 | 0.952 | 0.222 | 0.238 | 4 | No |
| Cognitive activity | Social support | H2PC | Disc3 | 0.955 | 0.765 | 0.965 | 0.664 | 0.965 | 0.664 | 16 | Yes |
| BMI | MIND diet | H2PC | Disc3 | 0.92 | 0.705 | 0.909 | 0.587 | 0.682 | 0.440 | 12 | Yes |
| BMI | Blood pressure^§^ | H2PC | Disc3 | 1 | 0.916 | 0.998 | 0.952 | 0.998 | 0.952 | 16 | Yes |
| Perceived stress | Anxiety | H2PC | Disc3 | 1 | 0.87 | 0.997 | 0.858 | 0.997 | 0.858 | 16 | Yes |
| Perceived stress | Resilient coping | H2PC | Disc3 | 0.979 | 0.642 | 0.949 | 0.621 | 0.356 | 0.233 | 6 | No |
| Chronic stress | Perceived stress | H2PC | Disc3 | 1 | 0.612 | 0.981 | 0.704 | 0.981 | 0.704 | 16 | Yes |
| Chronic stress | Anxiety | H2PC | Disc3 | 0.999 | 0.812 | 0.993 | 0.878 | 0.993 | 0.878 | 16 | Yes |
| Age | Chronic stress | H2PC | Disc3 | 0.953 | 1 | 0.993 | 1 | 0.993 | 1 | 16 | Yes |
| Gender | Cognitive activity | H2PC | Disc3 | 1 | 1 | 0.999 | 1 | 0.999 | 1 | 16 | Yes |
| Gender | MIND diet | H2PC | Disc3 | 0.959 | 1 | 0.963 | 1 | 0.963 | 1 | 16 | Yes |
| Gender | Alcohol | H2PC | Disc3 | 1 | 1 | 0.999 | 1 | 0.999 | 1 | 16 | Yes |
| Gender | Marital status | H2PC | Disc3 | 1 | 1 | 1 | 1 | 1 | 1 | 16 | Yes |
| IRSAD decile | Alcohol | H2PC | Disc3 | 0.941 | 0.767 | 0.93 | 0.707 | 0.697 | 0.53 | 12 | Yes |
| Education | Cognitive activity | H2PC | Disc3 | 0.999 | 1 | 1 | 1 | 1 | 1 | 16 | Yes |
| Education | IRSAD decile | H2PC | Disc3 | 0.979 | 1 | 0.965 | 1 | 0.905 | 0.938 | 15 | Yes |
| Social support | Physical activity | H2PC | Disc3 | 0.979 | 0.849 | 0.963 | 0.711 | 0.903 | 0.666 | 15 | Yes |
| Depression | Social support | H2PC | Disc3 | 0.982 | 0.829 | 0.984 | 0.838 | 0.984 | 0.838 | 16 | Yes |
| Anxiety | Depression | H2PC | Disc3 | 1 | 0.75 | 0.997 | 0.785 | 0.997 | 0.785 | 16 | Yes |
| Resilient coping | Cognitive activity | H2PC | Disc3 | 0.979 | 0.59 | 0.98 | 0.655 | 0.919 | 0.614 | 15 | Yes |
| Recent stressors | Perceived stress | H2PC | Disc3 | 1 | 1 | 1 | 1 | 1 | 1 | 16 | Yes |
| Recent stressors | Chronic stress | H2PC | Disc3 | 0.999 | 1 | 0.983 | 1 | 0.983 | 1 | 16 | Yes |
| Recent stressors | Marital status | H2PC | Disc3 | 0.927 | 1 | 0.93 | 1 | 0.465 | 0.5 | 8 | No |
| Physical activity | MIND diet | H2PC | Disc5 | 0.867 | 0.671 | 0.899 | 0.704 | 0.899 | 0.704 | 16 | Yes |
| Physical activity | BMI | H2PC | Disc5 | 0.999 | 0.554 | 0.973 | 0.563 | 0.912 | 0.528 | 15 | Yes |
| Cholesterol^§^ | Blood pressure^§^ | H2PC | Disc5 | 0.864 | 0.92 | 0.903 | 0.842 | 0.847 | 0.789 | 15 | Yes |
| Cholesterol^§^ | Diabetes^§^ | H2PC | Disc5 | 1 | 0.577 | 1 | 0.534 | 0.75 | 0.4 | 12 | Yes |
| Cognitive activity | Social support | H2PC | Disc5 | 0.989 | 0.697 | 0.965 | 0.664 | 0.965 | 0.664 | 16 | Yes |
| BMI | MIND diet | H2PC | Disc5 | 0.937 | 0.584 | 0.909 | 0.587 | 0.682 | 0.44 | 12 | Yes |
| BMI | Blood pressure^§^ | H2PC | Disc5 | 1 | 0.957 | 0.998 | 0.952 | 0.998 | 0.952 | 16 | Yes |
| Perceived stress | Anxiety | H2PC | Disc5 | 1 | 0.936 | 0.997 | 0.858 | 0.997 | 0.858 | 16 | Yes |
| Perceived stress | Resilient coping | H2PC | Disc5 | 0.89 | 0.523 | 0.949 | 0.621 | 0.356 | 0.233 | 6 | No |
| Chronic stress | Perceived stress | H2PC | Disc5 | 1 | 0.722 | 0.981 | 0.704 | 0.981 | 0.704 | 16 | Yes |
| Chronic stress | Anxiety | H2PC | Disc5 | 1 | 0.939 | 0.993 | 0.878 | 0.993 | 0.878 | 16 | Yes |
| Age | Blood pressure^§^ | H2PC | Disc5 | 0.863 | 1 | 0.952 | 1 | 0.833 | 0.875 | 14 | Yes |
| Age | Chronic stress | H2PC | Disc5 | 0.992 | 1 | 0.993 | 1 | 0.993 | 1 | 16 | Yes |
| Age | Marital status | H2PC | Disc5 | 0.967 | 1 | 0.964 | 1 | 0.723 | 0.75 | 12 | Yes |
| Gender | Cognitive activity | H2PC | Disc5 | 0.996 | 1 | 0.999 | 1 | 0.999 | 1 | 16 | Yes |
| Gender | MIND diet | H2PC | Disc5 | 0.933 | 1 | 0.963 | 1 | 0.963 | 1 | 16 | Yes |
| Gender | Alcohol | H2PC | Disc5 | 1 | 1 | 0.999 | 1 | 0.999 | 1 | 16 | Yes |
| Gender | Marital status | H2PC | Disc5 | 1 | 1 | 1 | 1 | 1 | 1 | 16 | Yes |
| IRSAD decile | Alcohol | H2PC | Disc5 | 0.925 | 0.719 | 0.93 | 0.707 | 0.697 | 0.53 | 12 | Yes |
| Education | Cognitive activity | H2PC | Disc5 | 0.999 | 1 | 1 | 1 | 1 | 1 | 16 | Yes |
| Education | IRSAD decile | H2PC | Disc5 | 0.978 | 1 | 0.965 | 1 | 0.905 | 0.938 | 15 | Yes |
| Social support | Physical activity | H2PC | Disc5 | 0.917 | 0.624 | 0.963 | 0.711 | 0.903 | 0.666 | 15 | Yes |
| Depression | Social support | H2PC | Disc5 | 0.983 | 0.908 | 0.984 | 0.838 | 0.984 | 0.838 | 16 | Yes |
| Anxiety | Depression | H2PC | Disc5 | 1 | 0.909 | 0.997 | 0.785 | 0.997 | 0.785 | 16 | Yes |
| Resilient coping | Cognitive activity | H2PC | Disc5 | 1 | 0.704 | 0.98 | 0.655 | 0.919 | 0.614 | 15 | Yes |
| Recent stressors | Perceived stress | H2PC | Disc5 | 1 | 1 | 1 | 1 | 1 | 1 | 16 | Yes |
| Recent stressors | Chronic stress | H2PC | Disc5 | 0.999 | 1 | 0.983 | 1 | 0.983 | 1 | 16 | Yes |
| Physical activity | MIND diet | H2PC | INT | 0.942 | 0.786 | 0.899 | 0.704 | 0.899 | 0.704 | 16 | Yes |
| Physical activity | BMI | H2PC | INT | 1 | 0.597 | 0.973 | 0.563 | 0.912 | 0.528 | 15 | Yes |
| Cholesterol^§^ | Blood pressure^§^ | H2PC | INT | 0.913 | 0.798 | 0.903 | 0.842 | 0.847 | 0.789 | 15 | Yes |
| Cognitive activity | Social support | H2PC | INT | 0.998 | 0.606 | 0.965 | 0.664 | 0.965 | 0.664 | 16 | Yes |
| Diabetes^§^ | Cholesterol^§^ | H2PC | INT | 1 | 0.518 | 0.881 | 0.515 | 0.22 | 0.129 | 4 | No |
| BMI | MIND diet | H2PC | INT | 0.91 | 0.532 | 0.909 | 0.587 | 0.682 | 0.44 | 12 | Yes |
| BMI | Blood pressure^§^ | H2PC | INT | 1 | 0.965 | 0.998 | 0.952 | 0.998 | 0.952 | 16 | Yes |
| Perceived stress | Anxiety | H2PC | INT | 1 | 0.952 | 0.997 | 0.858 | 0.997 | 0.858 | 16 | Yes |
| Chronic stress | Perceived stress | H2PC | INT | 1 | 0.696 | 0.981 | 0.704 | 0.981 | 0.704 | 16 | Yes |
| Chronic stress | Anxiety | H2PC | INT | 1 | 0.897 | 0.993 | 0.878 | 0.993 | 0.878 | 16 | Yes |
| Age | Blood pressure^§^ | H2PC | INT | 0.996 | 1 | 0.952 | 1 | 0.833 | 0.875 | 14 | Yes |
| Age | Chronic stress | H2PC | INT | 1 | 1 | 0.993 | 1 | 0.993 | 1 | 16 | Yes |
| Gender | Cognitive activity | H2PC | INT | 0.996 | 1 | 0.999 | 1 | 0.999 | 1 | 16 | Yes |
| Gender | MIND diet | H2PC | INT | 0.97 | 1 | 0.963 | 1 | 0.963 | 1 | 16 | Yes |
| Gender | Alcohol | H2PC | INT | 0.998 | 1 | 0.999 | 1 | 0.999 | 1 | 16 | Yes |
| Gender | Marital status | H2PC | INT | 1 | 1 | 1 | 1 | 1 | 1 | 16 | Yes |
| IRSAD decile | Alcohol | H2PC | INT | 0.945 | 0.698 | 0.93 | 0.707 | 0.697 | 0.53 | 12 | Yes |
| Education | Cognitive activity | H2PC | INT | 1 | 1 | 1 | 1 | 1 | 1 | 16 | Yes |
| Education | IRSAD decile | H2PC | INT | 0.98 | 1 | 0.965 | 1 | 0.905 | 0.938 | 15 | Yes |
| Social support | Physical activity | H2PC | INT | 0.973 | 0.754 | 0.963 | 0.711 | 0.903 | 0.666 | 15 | Yes |
| Depression | Social support | H2PC | INT | 0.998 | 0.868 | 0.984 | 0.838 | 0.984 | 0.838 | 16 | Yes |
| Anxiety | Depression | H2PC | INT | 1 | 0.947 | 0.997 | 0.785 | 0.997 | 0.785 | 16 | Yes |
| Resilient coping | Cognitive activity | H2PC | INT | 1 | 0.751 | 0.98 | 0.655 | 0.919 | 0.614 | 15 | Yes |
| Recent stressors | Perceived stress | H2PC | INT | 1 | 1 | 1 | 1 | 1 | 1 | 16 | Yes |
| Recent stressors | Chronic stress | H2PC | INT | 0.926 | 1 | 0.983 | 1 | 0.983 | 1 | 16 | Yes |
| Physical activity | MIND diet | H2PC | NPN | 0.855 | 0.706 | 0.899 | 0.704 | 0.899 | 0.704 | 16 | Yes |
| Physical activity | BMI | H2PC | NPN | 1 | 0.652 | 0.973 | 0.563 | 0.912 | 0.528 | 15 | Yes |
| Cholesterol^§^ | Blood pressure^§^ | H2PC | NPN | 0.914 | 0.881 | 0.903 | 0.842 | 0.847 | 0.789 | 15 | Yes |
| Cholesterol^§^ | Diabetes^§^ | H2PC | NPN | 1 | 0.52 | 1 | 0.534 | 0.75 | 0.4 | 12 | Yes |
| Cognitive activity | Social support | H2PC | NPN | 0.997 | 0.598 | 0.965 | 0.664 | 0.965 | 0.664 | 16 | Yes |
| MIND diet | BMI | H2PC | NPN | 0.955 | 0.547 | 0.971 | 0.552 | 0.243 | 0.138 | 4 | No |
| Alcohol | IRSAD decile | H2PC | NPN | 0.927 | 0.632 | 0.941 | 0.633 | 0.235 | 0.158 | 4 | No |
| BMI | Blood pressure^§^ | H2PC | NPN | 0.999 | 0.981 | 0.998 | 0.952 | 0.998 | 0.952 | 16 | Yes |
| Perceived stress | Anxiety | H2PC | NPN | 1 | 0.949 | 0.997 | 0.858 | 0.997 | 0.858 | 16 | Yes |
| Chronic stress | Perceived stress | H2PC | NPN | 1 | 0.712 | 0.981 | 0.704 | 0.981 | 0.704 | 16 | Yes |
| Chronic stress | Anxiety | H2PC | NPN | 1 | 0.92 | 0.993 | 0.878 | 0.993 | 0.878 | 16 | Yes |
| Age | Blood pressure^§^ | H2PC | NPN | 0.977 | 1 | 0.952 | 1 | 0.833 | 0.875 | 14 | Yes |
| Age | Chronic stress | H2PC | NPN | 1 | 1 | 0.993 | 1 | 0.993 | 1 | 16 | Yes |
| Age | Marital status | H2PC | NPN | 0.881 | 1 | 0.964 | 1 | 0.723 | 0.75 | 12 | Yes |
| Gender | Cognitive activity | H2PC | NPN | 0.997 | 1 | 0.999 | 1 | 0.999 | 1 | 16 | Yes |
| Gender | MIND diet | H2PC | NPN | 0.935 | 1 | 0.963 | 1 | 0.963 | 1 | 16 | Yes |
| Gender | Alcohol | H2PC | NPN | 0.999 | 1 | 0.999 | 1 | 0.999 | 1 | 16 | Yes |
| Gender | Marital status | H2PC | NPN | 1 | 1 | 1 | 1 | 1 | 1 | 16 | Yes |
| Education | Cognitive activity | H2PC | NPN | 1 | 1 | 1 | 1 | 1 | 1 | 16 | Yes |
| Social support | Physical activity | H2PC | NPN | 0.986 | 0.786 | 0.963 | 0.711 | 0.903 | 0.666 | 15 | Yes |
| Depression | Social support | H2PC | NPN | 0.995 | 0.861 | 0.984 | 0.838 | 0.984 | 0.838 | 16 | Yes |
| Anxiety | Depression | H2PC | NPN | 1 | 0.922 | 0.997 | 0.785 | 0.997 | 0.785 | 16 | Yes |
| Resilient coping | Cognitive activity | H2PC | NPN | 0.999 | 0.716 | 0.98 | 0.655 | 0.919 | 0.614 | 15 | Yes |
| Recent stressors | Perceived stress | H2PC | NPN | 1 | 1 | 1 | 1 | 1 | 1 | 16 | Yes |
| Recent stressors | Chronic stress | H2PC | NPN | 0.948 | 1 | 0.983 | 1 | 0.983 | 1 | 16 | Yes |
| Physical activity | MIND diet | HC | Disc3 | 0.911 | 0.634 | 0.899 | 0.704 | 0.899 | 0.704 | 16 | Yes |
| Cholesterol^§^ | Blood pressure^§^ | HC | Disc3 | 0.882 | 0.754 | 0.903 | 0.842 | 0.847 | 0.789 | 15 | Yes |
| Cholesterol^§^ | Diabetes^§^ | HC | Disc3 | 1 | 0.514 | 1 | 0.534 | 0.75 | 0.4 | 12 | Yes |
| Cognitive activity | MIND diet | HC | Disc3 | 0.903 | 0.944 | 0.887 | 0.952 | 0.222 | 0.238 | 4 | No |
| Cognitive activity | Social support | HC | Disc3 | 0.982 | 0.777 | 0.965 | 0.664 | 0.965 | 0.664 | 16 | Yes |
| Cognitive activity | Resilient coping | HC | Disc3 | 0.982 | 0.586 | 0.982 | 0.586 | 0.061 | 0.037 | 1 | No |
| BMI | Physical activity | HC | Disc3 | 0.999 | 0.544 | 0.999 | 0.544 | 0.062 | 0.034 | 1 | No |
| BMI | MIND diet | HC | Disc3 | 0.957 | 0.633 | 0.909 | 0.587 | 0.682 | 0.44 | 12 | Yes |
| BMI | Blood pressure^§^ | HC | Disc3 | 1 | 0.906 | 0.998 | 0.952 | 0.998 | 0.952 | 16 | Yes |
| Perceived stress | Anxiety | HC | Disc3 | 1 | 0.813 | 0.997 | 0.858 | 0.997 | 0.858 | 16 | Yes |
| Perceived stress | Resilient coping | HC | Disc3 | 0.993 | 0.654 | 0.949 | 0.621 | 0.356 | 0.233 | 6 | No |
| Chronic stress | Perceived stress | HC | Disc3 | 1 | 0.671 | 0.981 | 0.704 | 0.981 | 0.704 | 16 | Yes |
| Chronic stress | Anxiety | HC | Disc3 | 1 | 0.769 | 0.993 | 0.878 | 0.993 | 0.878 | 16 | Yes |
| Age | Blood pressure^§^ | HC | Disc3 | 0.897 | 1 | 0.952 | 1 | 0.833 | 0.875 | 14 | Yes |
| Age | Chronic stress | HC | Disc3 | 0.987 | 1 | 0.993 | 1 | 0.993 | 1 | 16 | Yes |
| Age | Marital status | HC | Disc3 | 0.979 | 1 | 0.964 | 1 | 0.723 | 0.75 | 12 | Yes |
| Age | Social support | HC | Disc3 | 0.851 | 1 | 0.863 | 1 | 0.162 | 0.188 | 3 | No |
| Gender | Cognitive activity | HC | Disc3 | 1 | 1 | 0.999 | 1 | 0.999 | 1 | 16 | Yes |
| Gender | MIND diet | HC | Disc3 | 0.973 | 1 | 0.963 | 1 | 0.963 | 1 | 16 | Yes |
| Gender | Alcohol | HC | Disc3 | 1 | 1 | 0.999 | 1 | 0.999 | 1 | 16 | Yes |
| Gender | Marital status | HC | Disc3 | 1 | 1 | 1 | 1 | 1 | 1 | 16 | Yes |
| IRSAD decile | Alcohol | HC | Disc3 | 0.955 | 0.738 | 0.93 | 0.707 | 0.697 | 0.53 | 12 | Yes |
| Education | Cognitive activity | HC | Disc3 | 1 | 1 | 1 | 1 | 1 | 1 | 16 | Yes |
| Education | IRSAD decile | HC | Disc3 | 0.985 | 1 | 0.965 | 1 | 0.905 | 0.938 | 15 | Yes |
| Social support | Physical activity | HC | Disc3 | 0.991 | 0.714 | 0.963 | 0.711 | 0.903 | 0.666 | 15 | Yes |
| Depression | Social support | HC | Disc3 | 0.991 | 0.755 | 0.984 | 0.838 | 0.984 | 0.838 | 16 | Yes |
| Anxiety | Depression | HC | Disc3 | 1 | 0.515 | 0.997 | 0.785 | 0.997 | 0.785 | 16 | Yes |
| Recent stressors | Perceived stress | HC | Disc3 | 1 | 1 | 1 | 1 | 1 | 1 | 16 | Yes |
| Recent stressors | Chronic stress | HC | Disc3 | 1 | 1 | 0.983 | 1 | 0.983 | 1 | 16 | Yes |
| Recent stressors | Marital status | HC | Disc3 | 0.97 | 1 | 0.93 | 1 | 0.465 | 0.5 | 8 | No |
| Recent stressors | Depression | HC | Disc3 | 0.861 | 1 | 0.876 | 1 | 0.274 | 0.313 | 5 | No |
| Physical activity | MIND diet | HC | Disc5 | 0.931 | 0.685 | 0.899 | 0.704 | 0.899 | 0.704 | 16 | Yes |
| Physical activity | BMI | HC | Disc5 | 0.999 | 0.524 | 0.973 | 0.563 | 0.912 | 0.528 | 15 | Yes |
| Physical activity | Social support | HC | Disc5 | 0.957 | 0.521 | 0.957 | 0.521 | 0.06 | 0.033 | 1 | No |
| Cholesterol^§^ | Blood pressure^§^ | HC | Disc5 | 0.925 | 0.915 | 0.903 | 0.842 | 0.847 | 0.789 | 15 | Yes |
| Cholesterol^§^ | Diabetes^§^ | HC | Disc5 | 1 | 0.527 | 1 | 0.534 | 0.75 | 0.4 | 12 | Yes |
| Cognitive activity | Social support | HC | Disc5 | 0.996 | 0.707 | 0.965 | 0.664 | 0.965 | 0.664 | 16 | Yes |
| BMI | MIND diet | HC | Disc5 | 0.966 | 0.565 | 0.909 | 0.587 | 0.682 | 0.44 | 12 | Yes |
| BMI | Blood pressure^§^ | HC | Disc5 | 1 | 0.955 | 0.998 | 0.952 | 0.998 | 0.952 | 16 | Yes |
| Perceived stress | Anxiety | HC | Disc5 | 1 | 0.733 | 0.997 | 0.858 | 0.997 | 0.858 | 16 | Yes |
| Chronic stress | Perceived stress | HC | Disc5 | 1 | 0.846 | 0.981 | 0.704 | 0.981 | 0.704 | 16 | Yes |
| Chronic stress | Depression | HC | Disc5 | 0.91 | 0.63 | 0.898 | 0.731 | 0.224 | 0.183 | 4 | No |
| Chronic stress | Anxiety | HC | Disc5 | 1 | 0.924 | 0.993 | 0.878 | 0.993 | 0.878 | 16 | Yes |
| Age | Blood pressure^§^ | HC | Disc5 | 0.941 | 1 | 0.952 | 1 | 0.833 | 0.875 | 14 | Yes |
| Age | Chronic stress | HC | Disc5 | 0.997 | 1 | 0.993 | 1 | 0.993 | 1 | 16 | Yes |
| Age | Marital status | HC | Disc5 | 0.999 | 1 | 0.964 | 1 | 0.723 | 0.75 | 12 | Yes |
| Age | Social support | HC | Disc5 | 0.862 | 1 | 0.863 | 1 | 0.162 | 0.188 | 3 | No |
| Gender | Cognitive activity | HC | Disc5 | 0.999 | 1 | 0.999 | 1 | 0.999 | 1 | 16 | Yes |
| Gender | MIND diet | HC | Disc5 | 0.972 | 1 | 0.963 | 1 | 0.963 | 1 | 16 | Yes |
| Gender | Alcohol | HC | Disc5 | 1 | 1 | 0.999 | 1 | 0.999 | 1 | 16 | Yes |
| Gender | Marital status | HC | Disc5 | 1 | 1 | 1 | 1 | 1 | 1 | 16 | Yes |
| IRSAD decile | Alcohol | HC | Disc5 | 0.942 | 0.647 | 0.93 | 0.707 | 0.697 | 0.53 | 12 | Yes |
| Education | Cognitive activity | HC | Disc5 | 1 | 1 | 1 | 1 | 1 | 1 | 16 | Yes |
| Education | IRSAD decile | HC | Disc5 | 0.985 | 1 | 0.965 | 1 | 0.905 | 0.938 | 15 | Yes |
| Social support | IRSAD decile | HC | Disc5 | 0.888 | 0.703 | 0.867 | 0.717 | 0.325 | 0.269 | 6 | No |
| Depression | Social support | HC | Disc5 | 0.992 | 0.84 | 0.984 | 0.838 | 0.984 | 0.838 | 16 | Yes |
| Anxiety | Depression | HC | Disc5 | 1 | 0.583 | 0.997 | 0.785 | 0.997 | 0.785 | 16 | Yes |
| Resilient coping | Cognitive activity | HC | Disc5 | 1 | 0.543 | 0.98 | 0.655 | 0.919 | 0.614 | 15 | Yes |
| Resilient coping | Perceived stress | HC | Disc5 | 0.975 | 0.546 | 0.933 | 0.616 | 0.233 | 0.154 | 4 | No |
| Recent stressors | Perceived stress | HC | Disc5 | 1 | 1 | 1 | 1 | 1 | 1 | 16 | Yes |
| Recent stressors | Chronic stress | HC | Disc5 | 1 | 1 | 0.983 | 1 | 0.983 | 1 | 16 | Yes |
| Recent stressors | Marital status | HC | Disc5 | 0.928 | 1 | 0.93 | 1 | 0.465 | 0.5 | 8 | No |
| Recent stressors | Depression | HC | Disc5 | 0.85 | 1 | 0.876 | 1 | 0.274 | 0.313 | 5 | No |
| Physical activity | MIND diet | HC | INT | 0.972 | 0.769 | 0.899 | 0.704 | 0.899 | 0.704 | 16 | Yes |
| Physical activity | BMI | HC | INT | 1 | 0.504 | 0.973 | 0.563 | 0.912 | 0.528 | 15 | Yes |
| Cholesterol^§^ | Blood pressure^§^ | HC | INT | 0.943 | 0.762 | 0.903 | 0.842 | 0.847 | 0.789 | 15 | Yes |
| Cognitive activity | Social support | HC | INT | 1 | 0.653 | 0.965 | 0.664 | 0.965 | 0.664 | 16 | Yes |
| Diabetes^§^ | Cholesterol^§^ | HC | INT | 1 | 0.505 | 0.881 | 0.515 | 0.22 | 0.129 | 4 | No |
| BMI | MIND diet | HC | INT | 0.95 | 0.504 | 0.909 | 0.587 | 0.682 | 0.44 | 12 | Yes |
| BMI | Blood pressure^§^ | HC | INT | 1 | 0.948 | 0.998 | 0.952 | 0.998 | 0.952 | 16 | Yes |
| Perceived stress | Anxiety | HC | INT | 1 | 0.732 | 0.997 | 0.858 | 0.997 | 0.858 | 16 | Yes |
| Chronic stress | Perceived stress | HC | INT | 1 | 0.734 | 0.981 | 0.704 | 0.981 | 0.704 | 16 | Yes |
| Chronic stress | Anxiety | HC | INT | 1 | 0.905 | 0.993 | 0.878 | 0.993 | 0.878 | 16 | Yes |
| Age | MIND diet | HC | INT | 0.926 | 1 | 0.9 | 1 | 0.225 | 0.25 | 4 | No |
| Age | Blood pressure^§^ | HC | INT | 0.998 | 1 | 0.952 | 1 | 0.833 | 0.875 | 14 | Yes |
| Age | Chronic stress | HC | INT | 1 | 1 | 0.993 | 1 | 0.993 | 1 | 16 | Yes |
| Age | Marital status | HC | INT | 0.972 | 1 | 0.964 | 1 | 0.723 | 0.75 | 12 | Yes |
| Gender | Cognitive activity | HC | INT | 0.999 | 1 | 0.999 | 1 | 0.999 | 1 | 16 | Yes |
| Gender | MIND diet | HC | INT | 0.988 | 1 | 0.963 | 1 | 0.963 | 1 | 16 | Yes |
| Gender | Alcohol | HC | INT | 0.999 | 1 | 0.999 | 1 | 0.999 | 1 | 16 | Yes |
| Gender | Marital status | HC | INT | 1 | 1 | 1 | 1 | 1 | 1 | 16 | Yes |
| IRSAD decile | Alcohol | HC | INT | 0.954 | 0.622 | 0.93 | 0.707 | 0.697 | 0.53 | 12 | Yes |
| IRSAD decile | BMI | HC | INT | 0.858 | 0.511 | 0.695 | 0.522 | 0.087 | 0.065 | 2 | No |
| Education | Cognitive activity | HC | INT | 1 | 1 | 1 | 1 | 1 | 1 | 16 | Yes |
| Education | IRSAD decile | HC | INT | 0.989 | 1 | 0.965 | 1 | 0.905 | 0.938 | 15 | Yes |
| Social support | Physical activity | HC | INT | 0.991 | 0.571 | 0.963 | 0.711 | 0.903 | 0.666 | 15 | Yes |
| Social support | IRSAD decile | HC | INT | 0.919 | 0.66 | 0.867 | 0.717 | 0.325 | 0.269 | 6 | No |
| Depression | Social support | HC | INT | 1 | 0.719 | 0.984 | 0.838 | 0.984 | 0.838 | 16 | Yes |
| Anxiety | Depression | HC | INT | 1 | 0.618 | 0.997 | 0.785 | 0.997 | 0.785 | 16 | Yes |
| Resilient coping | Cognitive activity | HC | INT | 1 | 0.643 | 0.98 | 0.655 | 0.919 | 0.614 | 15 | Yes |
| Resilient coping | Depression | HC | INT | 0.893 | 0.586 | 0.895 | 0.578 | 0.112 | 0.072 | 2 | No |
| Recent stressors | Perceived stress | HC | INT | 1 | 1 | 1 | 1 | 1 | 1 | 16 | Yes |
| Recent stressors | Chronic stress | HC | INT | 0.969 | 1 | 0.983 | 1 | 0.983 | 1 | 16 | Yes |
| Physical activity | MIND diet | HC | NPN | 0.926 | 0.693 | 0.899 | 0.704 | 0.899 | 0.704 | 16 | Yes |
| Physical activity | BMI | HC | NPN | 1 | 0.527 | 0.973 | 0.563 | 0.912 | 0.528 | 15 | Yes |
| Cholesterol^§^ | Blood pressure^§^ | HC | NPN | 0.948 | 0.868 | 0.903 | 0.842 | 0.847 | 0.789 | 15 | Yes |
| Cholesterol^§^ | Diabetes^§^ | HC | NPN | 1 | 0.515 | 1 | 0.534 | 0.75 | 0.4 | 12 | Yes |
| Cognitive activity | Social support | HC | NPN | 0.998 | 0.624 | 0.965 | 0.664 | 0.965 | 0.664 | 16 | Yes |
| MIND diet | BMI | HC | NPN | 0.977 | 0.541 | 0.971 | 0.552 | 0.243 | 0.138 | 4 | No |
| Alcohol | IRSAD decile | HC | NPN | 0.94 | 0.64 | 0.941 | 0.633 | 0.235 | 0.158 | 4 | No |
| BMI | Blood pressure^§^ | HC | NPN | 0.999 | 0.974 | 0.998 | 0.952 | 0.998 | 0.952 | 16 | Yes |
| BMI | IRSAD decile | HC | NPN | 0.899 | 0.605 | 0.893 | 0.614 | 0.168 | 0.115 | 3 | No |
| Perceived stress | Anxiety | HC | NPN | 1 | 0.679 | 0.997 | 0.858 | 0.997 | 0.858 | 16 | Yes |
| Chronic stress | Perceived stress | HC | NPN | 1 | 0.778 | 0.981 | 0.704 | 0.981 | 0.704 | 16 | Yes |
| Chronic stress | Depression | HC | NPN | 0.9 | 0.599 | 0.898 | 0.731 | 0.224 | 0.183 | 4 | No |
| Chronic stress | Anxiety | HC | NPN | 1 | 0.928 | 0.993 | 0.878 | 0.993 | 0.878 | 16 | Yes |
| Age | MIND diet | HC | NPN | 0.877 | 1 | 0.9 | 1 | 0.225 | 0.25 | 4 | No |
| Age | Blood pressure^§^ | HC | NPN | 0.99 | 1 | 0.952 | 1 | 0.833 | 0.875 | 14 | Yes |
| Age | Chronic stress | HC | NPN | 1 | 1 | 0.993 | 1 | 0.993 | 1 | 16 | Yes |
| Age | Marital status | HC | NPN | 0.988 | 1 | 0.964 | 1 | 0.723 | 0.75 | 12 | Yes |
| Gender | Cognitive activity | HC | NPN | 0.999 | 1 | 0.999 | 1 | 0.999 | 1 | 16 | Yes |
| Gender | MIND diet | HC | NPN | 0.974 | 1 | 0.963 | 1 | 0.963 | 1 | 16 | Yes |
| Gender | Alcohol | HC | NPN | 0.999 | 1 | 0.999 | 1 | 0.999 | 1 | 16 | Yes |
| Gender | Marital status | HC | NPN | 1 | 1 | 1 | 1 | 1 | 1 | 16 | Yes |
| Education | Cognitive activity | HC | NPN | 1 | 1 | 1 | 1 | 1 | 1 | 16 | Yes |
| Education | IRSAD decile | HC | NPN | 0.886 | 1 | 0.965 | 1 | 0.905 | 0.938 | 15 | Yes |
| Social support | Physical activity | HC | NPN | 0.993 | 0.595 | 0.963 | 0.711 | 0.903 | 0.666 | 15 | Yes |
| Depression | Social support | HC | NPN | 0.998 | 0.726 | 0.984 | 0.838 | 0.984 | 0.838 | 16 | Yes |
| Anxiety | Depression | HC | NPN | 1 | 0.561 | 0.997 | 0.785 | 0.997 | 0.785 | 16 | Yes |
| Resilient coping | Cognitive activity | HC | NPN | 1 | 0.648 | 0.98 | 0.655 | 0.919 | 0.614 | 15 | Yes |
| Resilient coping | Perceived stress | HC | NPN | 0.891 | 0.731 | 0.933 | 0.616 | 0.233 | 0.154 | 4 | No |
| Resilient coping | Chronic stress | HC | NPN | 0.888 | 0.618 | 0.883 | 0.62 | 0.11 | 0.078 | 2 | No |
| Recent stressors | Perceived stress | HC | NPN | 1 | 1 | 1 | 1 | 1 | 1 | 16 | Yes |
| Recent stressors | Chronic stress | HC | NPN | 0.984 | 1 | 0.983 | 1 | 0.983 | 1 | 16 | Yes |
| Recent stressors | Marital status | HC | NPN | 0.9 | 1 | 0.93 | 1 | 0.465 | 0.5 | 8 | No |
| Recent stressors | Depression | HC | NPN | 0.913 | 1 | 0.876 | 1 | 0.274 | 0.313 | 5 | No |
| Physical activity | MIND diet | MMHC | Disc5 | 0.893 | 0.625 | 0.899 | 0.704 | 0.899 | 0.704 | 16 | Yes |
| Physical activity | BMI | MMHC | Disc5 | 0.999 | 0.536 | 0.973 | 0.563 | 0.912 | 0.528 | 15 | Yes |
| Cholesterol^§^ | Blood pressure^§^ | MMHC | Disc5 | 0.884 | 0.926 | 0.903 | 0.842 | 0.847 | 0.789 | 15 | Yes |
| Cholesterol^§^ | Diabetes^§^ | MMHC | Disc5 | 1 | 0.577 | 1 | 0.534 | 0.75 | 0.4 | 12 | Yes |
| Cognitive activity | Social support | MMHC | Disc5 | 0.988 | 0.683 | 0.965 | 0.664 | 0.965 | 0.664 | 16 | Yes |
| BMI | MIND diet | MMHC | Disc5 | 0.962 | 0.57 | 0.909 | 0.587 | 0.682 | 0.44 | 12 | Yes |
| BMI | Blood pressure^§^ | MMHC | Disc5 | 1 | 0.962 | 0.998 | 0.952 | 0.998 | 0.952 | 16 | Yes |
| Perceived stress | Anxiety | MMHC | Disc5 | 1 | 0.925 | 0.997 | 0.858 | 0.997 | 0.858 | 16 | Yes |
| Perceived stress | Resilient coping | MMHC | Disc5 | 0.863 | 0.566 | 0.949 | 0.621 | 0.356 | 0.233 | 6 | No |
| Chronic stress | Perceived stress | MMHC | Disc5 | 1 | 0.709 | 0.981 | 0.704 | 0.981 | 0.704 | 16 | Yes |
| Chronic stress | Anxiety | MMHC | Disc5 | 1 | 0.935 | 0.993 | 0.878 | 0.993 | 0.878 | 16 | Yes |
| Age | Blood pressure^§^ | MMHC | Disc5 | 0.863 | 1 | 0.952 | 1 | 0.833 | 0.875 | 14 | Yes |
| Age | Chronic stress | MMHC | Disc5 | 0.997 | 1 | 0.993 | 1 | 0.993 | 1 | 16 | Yes |
| Age | Marital status | MMHC | Disc5 | 0.966 | 1 | 0.964 | 1 | 0.723 | 0.75 | 12 | Yes |
| Gender | Cognitive activity | MMHC | Disc5 | 0.999 | 1 | 0.999 | 1 | 0.999 | 1 | 16 | Yes |
| Gender | MIND diet | MMHC | Disc5 | 0.924 | 1 | 0.963 | 1 | 0.963 | 1 | 16 | Yes |
| Gender | Alcohol | MMHC | Disc5 | 1 | 1 | 0.999 | 1 | 0.999 | 1 | 16 | Yes |
| Gender | Marital status | MMHC | Disc5 | 0.999 | 1 | 1 | 1 | 1 | 1 | 16 | Yes |
| IRSAD decile | Alcohol | MMHC | Disc5 | 0.955 | 0.74 | 0.93 | 0.707 | 0.697 | 0.53 | 12 | Yes |
| Education | Cognitive activity | MMHC | Disc5 | 0.999 | 1 | 1 | 1 | 1 | 1 | 16 | Yes |
| Education | IRSAD decile | MMHC | Disc5 | 0.985 | 1 | 0.965 | 1 | 0.905 | 0.938 | 15 | Yes |
| Social support | Physical activity | MMHC | Disc5 | 0.939 | 0.663 | 0.963 | 0.711 | 0.903 | 0.666 | 15 | Yes |
| Social support | IRSAD decile | MMHC | Disc5 | 0.858 | 0.746 | 0.867 | 0.717 | 0.325 | 0.269 | 6 | No |
| Depression | Social support | MMHC | Disc5 | 0.987 | 0.902 | 0.984 | 0.838 | 0.984 | 0.838 | 16 | Yes |
| Anxiety | Depression | MMHC | Disc5 | 1 | 0.909 | 0.997 | 0.785 | 0.997 | 0.785 | 16 | Yes |
| Resilient coping | Cognitive activity | MMHC | Disc5 | 1 | 0.705 | 0.98 | 0.655 | 0.919 | 0.614 | 15 | Yes |
| Recent stressors | Perceived stress | MMHC | Disc5 | 1 | 1 | 1 | 1 | 1 | 1 | 16 | Yes |
| Recent stressors | Chronic stress | MMHC | Disc5 | 1 | 1 | 0.983 | 1 | 0.983 | 1 | 16 | Yes |
| Physical activity | MIND diet | MMHC | Disc3 | 0.89 | 0.683 | 0.899 | 0.704 | 0.899 | 0.704 | 16 | Yes |
| Physical activity | BMI | MMHC | Disc3 | 0.999 | 0.567 | 0.973 | 0.563 | 0.912 | 0.528 | 15 | Yes |
| Cholesterol^§^ | Blood pressure^§^ | MMHC | Disc3 | 0.858 | 0.781 | 0.903 | 0.842 | 0.847 | 0.789 | 15 | Yes |
| Cholesterol^§^ | Diabetes^§^ | MMHC | Disc3 | 1 | 0.558 | 1 | 0.534 | 0.75 | 0.4 | 12 | Yes |
| Cognitive activity | MIND diet | MMHC | Disc3 | 0.87 | 0.954 | 0.887 | 0.952 | 0.222 | 0.238 | 4 | No |
| Cognitive activity | Social support | MMHC | Disc3 | 0.962 | 0.775 | 0.965 | 0.664 | 0.965 | 0.664 | 16 | Yes |
| BMI | MIND diet | MMHC | Disc3 | 0.947 | 0.702 | 0.909 | 0.587 | 0.682 | 0.44 | 12 | Yes |
| BMI | Blood pressure^§^ | MMHC | Disc3 | 1 | 0.925 | 0.998 | 0.952 | 0.998 | 0.952 | 16 | Yes |
| Perceived stress | Anxiety | MMHC | Disc3 | 1 | 0.874 | 0.997 | 0.858 | 0.997 | 0.858 | 16 | Yes |
| Perceived stress | Resilient coping | MMHC | Disc3 | 0.978 | 0.651 | 0.949 | 0.621 | 0.356 | 0.233 | 6 | No |
| Chronic stress | Perceived stress | MMHC | Disc3 | 1 | 0.613 | 0.981 | 0.704 | 0.981 | 0.704 | 16 | Yes |
| Chronic stress | Anxiety | MMHC | Disc3 | 1 | 0.81 | 0.993 | 0.878 | 0.993 | 0.878 | 16 | Yes |
| Age | Chronic stress | MMHC | Disc3 | 0.978 | 1 | 0.993 | 1 | 0.993 | 1 | 16 | Yes |
| Gender | Cognitive activity | MMHC | Disc3 | 1 | 1 | 0.999 | 1 | 0.999 | 1 | 16 | Yes |
| Gender | MIND diet | MMHC | Disc3 | 0.954 | 1 | 0.963 | 1 | 0.963 | 1 | 16 | Yes |
| Gender | Alcohol | MMHC | Disc3 | 1 | 1 | 0.999 | 1 | 0.999 | 1 | 16 | Yes |
| Gender | Marital status | MMHC | Disc3 | 0.999 | 1 | 1 | 1 | 1 | 1 | 16 | Yes |
| IRSAD decile | Alcohol | MMHC | Disc3 | 0.965 | 0.778 | 0.93 | 0.707 | 0.697 | 0.53 | 12 | Yes |
| Education | Cognitive activity | MMHC | Disc3 | 0.999 | 1 | 1 | 1 | 1 | 1 | 16 | Yes |
| Education | IRSAD decile | MMHC | Disc3 | 0.987 | 1 | 0.965 | 1 | 0.905 | 0.938 | 15 | Yes |
| Social support | Physical activity | MMHC | Disc3 | 0.988 | 0.852 | 0.963 | 0.711 | 0.903 | 0.666 | 15 | Yes |
| Depression | Social support | MMHC | Disc3 | 0.992 | 0.838 | 0.984 | 0.838 | 0.984 | 0.838 | 16 | Yes |
| Anxiety | Depression | MMHC | Disc3 | 1 | 0.722 | 0.997 | 0.785 | 0.997 | 0.785 | 16 | Yes |
| Resilient coping | Cognitive activity | MMHC | Disc3 | 0.99 | 0.595 | 0.98 | 0.655 | 0.919 | 0.614 | 15 | Yes |
| Recent stressors | Perceived stress | MMHC | Disc3 | 1 | 1 | 1 | 1 | 1 | 1 | 16 | Yes |
| Recent stressors | Chronic stress | MMHC | Disc3 | 0.999 | 1 | 0.983 | 1 | 0.983 | 1 | 16 | Yes |
| Recent stressors | Marital status | MMHC | Disc3 | 0.926 | 1 | 0.93 | 1 | 0.465 | 0.5 | 8 | No |
| Physical activity | MIND diet | MMHC | INT | 0.753 | 0.753 | 0.899 | 0.704 | 0.899 | 0.704 | 16 | Yes |
| Physical activity | BMI | MMHC | INT | 0.596 | 0.596 | 0.973 | 0.563 | 0.912 | 0.528 | 15 | Yes |
| Cholesterol^§^ | Blood pressure^§^ | MMHC | INT | 0.804 | 0.804 | 0.903 | 0.842 | 0.847 | 0.789 | 15 | Yes |
| Cognitive activity | Social support | MMHC | INT | 0.603 | 0.603 | 0.965 | 0.664 | 0.965 | 0.664 | 16 | Yes |
| Diabetes^§^ | Cholesterol^§^ | MMHC | INT | 0.526 | 0.526 | 0.881 | 0.515 | 0.22 | 0.129 | 4 | No |
| BMI | MIND diet | MMHC | INT | 0.503 | 0.503 | 0.909 | 0.587 | 0.682 | 0.44 | 12 | Yes |
| BMI | Blood pressure^§^ | MMHC | INT | 0.974 | 0.974 | 0.998 | 0.952 | 0.998 | 0.952 | 16 | Yes |
| Perceived stress | Anxiety | MMHC | INT | 0.947 | 0.947 | 0.997 | 0.858 | 0.997 | 0.858 | 16 | Yes |
| Chronic stress | Perceived stress | MMHC | INT | 0.702 | 0.702 | 0.981 | 0.704 | 0.981 | 0.704 | 16 | Yes |
| Chronic stress | Anxiety | MMHC | INT | 0.894 | 0.894 | 0.993 | 0.878 | 0.993 | 0.878 | 16 | Yes |
| Age | Blood pressure^§^ | MMHC | INT | 1 | 1 | 0.952 | 1 | 0.833 | 0.875 | 14 | Yes |
| Age | Chronic stress | MMHC | INT | 1 | 1 | 0.993 | 1 | 0.993 | 1 | 16 | Yes |
| Gender | Cognitive activity | MMHC | INT | 1 | 1 | 0.999 | 1 | 0.999 | 1 | 16 | Yes |
| Gender | MIND diet | MMHC | INT | 1 | 1 | 0.963 | 1 | 0.963 | 1 | 16 | Yes |
| Gender | Alcohol | MMHC | INT | 1 | 1 | 0.999 | 1 | 0.999 | 1 | 16 | Yes |
| Gender | Marital status | MMHC | INT | 1 | 1 | 1 | 1 | 1 | 1 | 16 | Yes |
| IRSAD decile | Alcohol | MMHC | INT | 0.728 | 0.728 | 0.93 | 0.707 | 0.697 | 0.53 | 12 | Yes |
| IRSAD decile | BMI | MMHC | INT | 0.532 | 0.532 | 0.695 | 0.522 | 0.087 | 0.065 | 2 | No |
| Education | Cognitive activity | MMHC | INT | 1 | 1 | 1 | 1 | 1 | 1 | 16 | Yes |
| Education | IRSAD decile | MMHC | INT | 1 | 1 | 0.965 | 1 | 0.905 | 0.938 | 15 | Yes |
| Social support | Physical activity | MMHC | INT | 0.772 | 0.772 | 0.963 | 0.711 | 0.903 | 0.666 | 15 | Yes |
| Social support | IRSAD decile | MMHC | INT | 0.736 | 0.736 | 0.867 | 0.717 | 0.325 | 0.269 | 6 | No |
| Depression | Social support | MMHC | INT | 0.843 | 0.843 | 0.984 | 0.838 | 0.984 | 0.838 | 16 | Yes |
| Anxiety | Depression | MMHC | INT | 0.946 | 0.946 | 0.997 | 0.785 | 0.997 | 0.785 | 16 | Yes |
| Resilient coping | Cognitive activity | MMHC | INT | 0.749 | 0.749 | 0.98 | 0.655 | 0.919 | 0.614 | 15 | Yes |
| Recent stressors | Perceived stress | MMHC | INT | 1 | 1 | 1 | 1 | 1 | 1 | 16 | Yes |
| Recent stressors | Chronic stress | MMHC | INT | 1 | 1 | 0.983 | 1 | 0.983 | 1 | 16 | Yes |
| Physical activity | MIND diet | MMHC | NPN | 0.868 | 0.678 | 0.899 | 0.704 | 0.899 | 0.704 | 16 | Yes |
| Physical activity | BMI | MMHC | NPN | 1 | 0.653 | 0.973 | 0.563 | 0.912 | 0.528 | 15 | Yes |
| Cholesterol^§^ | Blood pressure^§^ | MMHC | NPN | 0.932 | 0.89 | 0.903 | 0.842 | 0.847 | 0.789 | 15 | Yes |
| Cholesterol^§^ | Diabetes^§^ | MMHC | NPN | 1 | 0.507 | 1 | 0.534 | 0.75 | 0.4 | 12 | Yes |
| Cognitive activity | Social support | MMHC | NPN | 0.998 | 0.567 | 0.965 | 0.664 | 0.965 | 0.664 | 16 | Yes |
| MIND diet | BMI | MMHC | NPN | 0.977 | 0.572 | 0.971 | 0.552 | 0.243 | 0.138 | 4 | No |
| Alcohol | IRSAD decile | MMHC | NPN | 0.955 | 0.61 | 0.941 | 0.633 | 0.235 | 0.158 | 4 | No |
| BMI | Blood pressure^§^ | MMHC | NPN | 0.999 | 0.984 | 0.998 | 0.952 | 0.998 | 0.952 | 16 | Yes |
| BMI | IRSAD decile | MMHC | NPN | 0.887 | 0.606 | 0.893 | 0.614 | 0.168 | 0.115 | 3 | No |
| Perceived stress | Anxiety | MMHC | NPN | 1 | 0.944 | 0.997 | 0.858 | 0.997 | 0.858 | 16 | Yes |
| Chronic stress | Perceived stress | MMHC | NPN | 1 | 0.7 | 0.981 | 0.704 | 0.981 | 0.704 | 16 | Yes |
| Chronic stress | Anxiety | MMHC | NPN | 1 | 0.917 | 0.993 | 0.878 | 0.993 | 0.878 | 16 | Yes |
| Age | Blood pressure^§^ | MMHC | NPN | 0.978 | 1 | 0.952 | 1 | 0.833 | 0.875 | 14 | Yes |
| Age | Chronic stress | MMHC | NPN | 1 | 1 | 0.993 | 1 | 0.993 | 1 | 16 | Yes |
| Age | Marital status | MMHC | NPN | 0.879 | 1 | 0.964 | 1 | 0.723 | 0.75 | 12 | Yes |
| Gender | Cognitive activity | MMHC | NPN | 0.999 | 1 | 0.999 | 1 | 0.999 | 1 | 16 | Yes |
| Gender | MIND diet | MMHC | NPN | 0.927 | 1 | 0.963 | 1 | 0.963 | 1 | 16 | Yes |
| Gender | Alcohol | MMHC | NPN | 0.999 | 1 | 0.999 | 1 | 0.999 | 1 | 16 | Yes |
| Gender | Marital status | MMHC | NPN | 0.999 | 1 | 1 | 1 | 1 | 1 | 16 | Yes |
| Education | Cognitive activity | MMHC | NPN | 1 | 1 | 1 | 1 | 1 | 1 | 16 | Yes |
| Education | IRSAD decile | MMHC | NPN | 0.88 | 1 | 0.965 | 1 | 0.905 | 0.938 | 15 | Yes |
| Social support | Physical activity | MMHC | NPN | 0.991 | 0.791 | 0.963 | 0.711 | 0.903 | 0.666 | 15 | Yes |
| Depression | Social support | MMHC | NPN | 0.997 | 0.837 | 0.984 | 0.838 | 0.984 | 0.838 | 16 | Yes |
| Anxiety | Depression | MMHC | NPN | 1 | 0.923 | 0.997 | 0.785 | 0.997 | 0.785 | 16 | Yes |
| Resilient coping | Cognitive activity | MMHC | NPN | 0.999 | 0.708 | 0.98 | 0.655 | 0.919 | 0.614 | 15 | Yes |
| Recent stressors | Perceived stress | MMHC | NPN | 1 | 1 | 1 | 1 | 1 | 1 | 16 | Yes |
| Recent stressors | Chronic stress | MMHC | NPN | 0.957 | 1 | 0.983 | 1 | 0.983 | 1 | 16 | Yes |
| Physical activity | MIND diet | TABU | Disc5 | 0.921 | 0.692 | 0.899 | 0.704 | 0.899 | 0.704 | 16 | Yes |
| Physical activity | BMI | TABU | Disc5 | 1 | 0.536 | 0.973 | 0.563 | 0.912 | 0.528 | 15 | Yes |
| Cholesterol^§^ | Blood pressure^§^ | TABU | Disc5 | 0.913 | 0.915 | 0.903 | 0.842 | 0.847 | 0.789 | 15 | Yes |
| Cholesterol^§^ | Diabetes^§^ | TABU | Disc5 | 1 | 0.531 | 1 | 0.534 | 0.75 | 0.4 | 12 | Yes |
| Cognitive activity | Social support | TABU | Disc5 | 0.995 | 0.694 | 0.965 | 0.664 | 0.965 | 0.664 | 16 | Yes |
| BMI | MIND diet | TABU | Disc5 | 0.962 | 0.57 | 0.909 | 0.587 | 0.682 | 0.44 | 12 | Yes |
| BMI | Blood pressure^§^ | TABU | Disc5 | 1 | 0.95 | 0.998 | 0.952 | 0.998 | 0.952 | 16 | Yes |
| Perceived stress | Anxiety | TABU | Disc5 | 1 | 0.845 | 0.997 | 0.858 | 0.997 | 0.858 | 16 | Yes |
| Chronic stress | Perceived stress | TABU | Disc5 | 1 | 0.769 | 0.981 | 0.704 | 0.981 | 0.704 | 16 | Yes |
| Chronic stress | Depression | TABU | Disc5 | 0.897 | 0.841 | 0.898 | 0.731 | 0.224 | 0.183 | 4 | No |
| Chronic stress | Anxiety | TABU | Disc5 | 1 | 0.893 | 0.993 | 0.878 | 0.993 | 0.878 | 16 | Yes |
| Age | Blood pressure^§^ | TABU | Disc5 | 0.939 | 1 | 0.952 | 1 | 0.833 | 0.875 | 14 | Yes |
| Age | Chronic stress | TABU | Disc5 | 0.998 | 1 | 0.993 | 1 | 0.993 | 1 | 16 | Yes |
| Age | Marital status | TABU | Disc5 | 0.998 | 1 | 0.964 | 1 | 0.723 | 0.75 | 12 | Yes |
| Age | Social support | TABU | Disc5 | 0.875 | 1 | 0.863 | 1 | 0.162 | 0.188 | 3 | No |
| Gender | Cognitive activity | TABU | Disc5 | 0.999 | 1 | 0.999 | 1 | 0.999 | 1 | 16 | Yes |
| Gender | MIND diet | TABU | Disc5 | 0.973 | 1 | 0.963 | 1 | 0.963 | 1 | 16 | Yes |
| Gender | Alcohol | TABU | Disc5 | 1 | 1 | 0.999 | 1 | 0.999 | 1 | 16 | Yes |
| Gender | Marital status | TABU | Disc5 | 1 | 1 | 1 | 1 | 1 | 1 | 16 | Yes |
| IRSAD decile | Alcohol | TABU | Disc5 | 0.94 | 0.667 | 0.93 | 0.707 | 0.697 | 0.53 | 12 | Yes |
| Education | Cognitive activity | TABU | Disc5 | 0.999 | 1 | 1 | 1 | 1 | 1 | 16 | Yes |
| Education | IRSAD decile | TABU | Disc5 | 0.985 | 1 | 0.965 | 1 | 0.905 | 0.938 | 15 | Yes |
| Social support | Physical activity | TABU | Disc5 | 0.955 | 0.547 | 0.963 | 0.711 | 0.903 | 0.666 | 15 | Yes |
| Social support | IRSAD decile | TABU | Disc5 | 0.885 | 0.754 | 0.867 | 0.717 | 0.325 | 0.269 | 6 | No |
| Depression | Social support | TABU | Disc5 | 0.993 | 0.923 | 0.984 | 0.838 | 0.984 | 0.838 | 16 | Yes |
| Anxiety | Depression | TABU | Disc5 | 1 | 0.847 | 0.997 | 0.785 | 0.997 | 0.785 | 16 | Yes |
| Resilient coping | Cognitive activity | TABU | Disc5 | 1 | 0.602 | 0.98 | 0.655 | 0.919 | 0.614 | 15 | Yes |
| Resilient coping | Perceived stress | TABU | Disc5 | 0.973 | 0.502 | 0.933 | 0.616 | 0.233 | 0.154 | 4 | No |
| Recent stressors | Perceived stress | TABU | Disc5 | 1 | 1 | 1 | 1 | 1 | 1 | 16 | Yes |
| Recent stressors | Chronic stress | TABU | Disc5 | 1 | 1 | 0.983 | 1 | 0.983 | 1 | 16 | Yes |
| Recent stressors | Marital status | TABU | Disc5 | 0.925 | 1 | 0.93 | 1 | 0.465 | 0.5 | 8 | No |
| Physical activity | MIND diet | TABU | Disc3 | 0.905 | 0.689 | 0.899 | 0.704 | 0.899 | 0.704 | 16 | Yes |
| Physical activity | BMI | TABU | Disc3 | 0.999 | 0.506 | 0.973 | 0.563 | 0.912 | 0.528 | 15 | Yes |
| Cholesterol^§^ | Blood pressure^§^ | TABU | Disc3 | 0.878 | 0.764 | 0.903 | 0.842 | 0.847 | 0.789 | 15 | Yes |
| Cholesterol^§^ | Diabetes^§^ | TABU | Disc3 | 1 | 0.51 | 1 | 0.534 | 0.75 | 0.4 | 12 | Yes |
| Cognitive activity | MIND diet | TABU | Disc3 | 0.906 | 0.948 | 0.887 | 0.952 | 0.222 | 0.238 | 4 | No |
| Cognitive activity | Social support | TABU | Disc3 | 0.978 | 0.716 | 0.965 | 0.664 | 0.965 | 0.664 | 16 | Yes |
| BMI | MIND diet | TABU | Disc3 | 0.95 | 0.665 | 0.909 | 0.587 | 0.682 | 0.44 | 12 | Yes |
| BMI | Blood pressure^§^ | TABU | Disc3 | 1 | 0.906 | 0.998 | 0.952 | 0.998 | 0.952 | 16 | Yes |
| Perceived stress | Anxiety | TABU | Disc3 | 1 | 0.799 | 0.997 | 0.858 | 0.997 | 0.858 | 16 | Yes |
| Perceived stress | Resilient coping | TABU | Disc3 | 0.993 | 0.687 | 0.949 | 0.621 | 0.356 | 0.233 | 6 | No |
| Chronic stress | Perceived stress | TABU | Disc3 | 1 | 0.652 | 0.981 | 0.704 | 0.981 | 0.704 | 16 | Yes |
| Chronic stress | Anxiety | TABU | Disc3 | 1 | 0.755 | 0.993 | 0.878 | 0.993 | 0.878 | 16 | Yes |
| Age | Blood pressure^§^ | TABU | Disc3 | 0.9 | 1 | 0.952 | 1 | 0.833 | 0.875 | 14 | Yes |
| Age | Chronic stress | TABU | Disc3 | 0.987 | 1 | 0.993 | 1 | 0.993 | 1 | 16 | Yes |
| Age | Marital status | TABU | Disc3 | 0.981 | 1 | 0.964 | 1 | 0.723 | 0.75 | 12 | Yes |
| Gender | Cognitive activity | TABU | Disc3 | 1 | 1 | 0.999 | 1 | 0.999 | 1 | 16 | Yes |
| Gender | MIND diet | TABU | Disc3 | 0.974 | 1 | 0.963 | 1 | 0.963 | 1 | 16 | Yes |
| Gender | Alcohol | TABU | Disc3 | 1 | 1 | 0.999 | 1 | 0.999 | 1 | 16 | Yes |
| Gender | Marital status | TABU | Disc3 | 1 | 1 | 1 | 1 | 1 | 1 | 16 | Yes |
| IRSAD decile | Alcohol | TABU | Disc3 | 0.954 | 0.735 | 0.93 | 0.707 | 0.697 | 0.53 | 12 | Yes |
| Education | Cognitive activity | TABU | Disc3 | 1 | 1 | 1 | 1 | 1 | 1 | 16 | Yes |
| Education | IRSAD decile | TABU | Disc3 | 0.987 | 1 | 0.965 | 1 | 0.905 | 0.938 | 15 | Yes |
| Social support | Physical activity | TABU | Disc3 | 0.991 | 0.795 | 0.963 | 0.711 | 0.903 | 0.666 | 15 | Yes |
| Depression | Social support | TABU | Disc3 | 0.993 | 0.87 | 0.984 | 0.838 | 0.984 | 0.838 | 16 | Yes |
| Anxiety | Depression | TABU | Disc3 | 1 | 0.657 | 0.997 | 0.785 | 0.997 | 0.785 | 16 | Yes |
| Resilient coping | Cognitive activity | TABU | Disc3 | 0.983 | 0.526 | 0.98 | 0.655 | 0.919 | 0.614 | 15 | Yes |
| Recent stressors | Perceived stress | TABU | Disc3 | 1 | 1 | 1 | 1 | 1 | 1 | 16 | Yes |
| Recent stressors | Chronic stress | TABU | Disc3 | 0.999 | 1 | 0.983 | 1 | 0.983 | 1 | 16 | Yes |
| Recent stressors | Marital status | TABU | Disc3 | 0.972 | 1 | 0.93 | 1 | 0.465 | 0.5 | 8 | No |
| Recent stressors | Depression | TABU | Disc3 | 0.854 | 1 | 0.876 | 1 | 0.274 | 0.313 | 5 | No |
| Physical activity | MIND diet | TABU | INT | 0.968 | 0.78 | 0.899 | 0.704 | 0.899 | 0.704 | 16 | Yes |
| Physical activity | BMI | TABU | INT | 1 | 0.534 | 0.973 | 0.563 | 0.912 | 0.528 | 15 | Yes |
| Cholesterol^§^ | Blood pressure^§^ | TABU | INT | 0.943 | 0.779 | 0.903 | 0.842 | 0.847 | 0.789 | 15 | Yes |
| Cognitive activity | Social support | TABU | INT | 1 | 0.593 | 0.965 | 0.664 | 0.965 | 0.664 | 16 | Yes |
| Diabetes^§^ | Cholesterol^§^ | TABU | INT | 1 | 0.51 | 0.881 | 0.515 | 0.22 | 0.129 | 4 | No |
| BMI | MIND diet | TABU | INT | 0.945 | 0.507 | 0.909 | 0.587 | 0.682 | 0.44 | 12 | Yes |
| BMI | Blood pressure^§^ | TABU | INT | 1 | 0.952 | 0.998 | 0.952 | 0.998 | 0.952 | 16 | Yes |
| Perceived stress | Anxiety | TABU | INT | 1 | 0.876 | 0.997 | 0.858 | 0.997 | 0.858 | 16 | Yes |
| Chronic stress | Perceived stress | TABU | INT | 1 | 0.658 | 0.981 | 0.704 | 0.981 | 0.704 | 16 | Yes |
| Chronic stress | Anxiety | TABU | INT | 1 | 0.864 | 0.993 | 0.878 | 0.993 | 0.878 | 16 | Yes |
| Age | MIND diet | TABU | INT | 0.925 | 1 | 0.9 | 1 | 0.225 | 0.25 | 4 | No |
| Age | Blood pressure^§^ | TABU | INT | 0.998 | 1 | 0.952 | 1 | 0.833 | 0.875 | 14 | Yes |
| Age | Chronic stress | TABU | INT | 1 | 1 | 0.993 | 1 | 0.993 | 1 | 16 | Yes |
| Age | Marital status | TABU | INT | 0.974 | 1 | 0.964 | 1 | 0.723 | 0.75 | 12 | Yes |
| Gender | Cognitive activity | TABU | INT | 0.999 | 1 | 0.999 | 1 | 0.999 | 1 | 16 | Yes |
| Gender | MIND diet | TABU | INT | 0.988 | 1 | 0.963 | 1 | 0.963 | 1 | 16 | Yes |
| Gender | Alcohol | TABU | INT | 0.999 | 1 | 0.999 | 1 | 0.999 | 1 | 16 | Yes |
| Gender | Marital status | TABU | INT | 1 | 1 | 1 | 1 | 1 | 1 | 16 | Yes |
| IRSAD decile | Alcohol | TABU | INT | 0.957 | 0.642 | 0.93 | 0.707 | 0.697 | 0.53 | 12 | Yes |
| Education | Cognitive activity | TABU | INT | 1 | 1 | 1 | 1 | 1 | 1 | 16 | Yes |
| Education | IRSAD decile | TABU | INT | 0.988 | 1 | 0.965 | 1 | 0.905 | 0.938 | 15 | Yes |
| Social support | Physical activity | TABU | INT | 0.99 | 0.66 | 0.963 | 0.711 | 0.903 | 0.666 | 15 | Yes |
| Social support | IRSAD decile | TABU | INT | 0.918 | 0.705 | 0.867 | 0.717 | 0.325 | 0.269 | 6 | No |
| Depression | Social support | TABU | INT | 0.999 | 0.845 | 0.984 | 0.838 | 0.984 | 0.838 | 16 | Yes |
| Anxiety | Depression | TABU | INT | 1 | 0.88 | 0.997 | 0.785 | 0.997 | 0.785 | 16 | Yes |
| Resilient coping | Cognitive activity | TABU | INT | 1 | 0.674 | 0.98 | 0.655 | 0.919 | 0.614 | 15 | Yes |
| Resilient coping | Depression | TABU | INT | 0.897 | 0.57 | 0.895 | 0.578 | 0.112 | 0.072 | 2 | No |
| Recent stressors | Perceived stress | TABU | INT | 1 | 1 | 1 | 1 | 1 | 1 | 16 | Yes |
| Recent stressors | Chronic stress | TABU | INT | 0.972 | 1 | 0.983 | 1 | 0.983 | 1 | 16 | Yes |
| Physical activity | MIND diet | TABU | NPN | 0.917 | 0.71 | 0.899 | 0.704 | 0.899 | 0.704 | 16 | Yes |
| Physical activity | BMI | TABU | NPN | 1 | 0.582 | 0.973 | 0.563 | 0.912 | 0.528 | 15 | Yes |
| Cholesterol^§^ | Blood pressure^§^ | TABU | NPN | 0.945 | 0.875 | 0.903 | 0.842 | 0.847 | 0.789 | 15 | Yes |
| Cholesterol^§^ | Diabetes^§^ | TABU | NPN | 1 | 0.518 | 1 | 0.534 | 0.75 | 0.4 | 12 | Yes |
| Cognitive activity | Social support | TABU | NPN | 0.999 | 0.572 | 0.965 | 0.664 | 0.965 | 0.664 | 16 | Yes |
| MIND diet | BMI | TABU | NPN | 0.977 | 0.549 | 0.971 | 0.552 | 0.243 | 0.138 | 4 | No |
| Alcohol | IRSAD decile | TABU | NPN | 0.941 | 0.651 | 0.941 | 0.633 | 0.235 | 0.158 | 4 | No |
| BMI | Blood pressure^§^ | TABU | NPN | 1 | 0.975 | 0.998 | 0.952 | 0.998 | 0.952 | 16 | Yes |
| BMI | IRSAD decile | TABU | NPN | 0.894 | 0.631 | 0.893 | 0.614 | 0.168 | 0.115 | 3 | No |
| Perceived stress | Anxiety | TABU | NPN | 1 | 0.856 | 0.997 | 0.858 | 0.997 | 0.858 | 16 | Yes |
| Chronic stress | Perceived stress | TABU | NPN | 1 | 0.689 | 0.981 | 0.704 | 0.981 | 0.704 | 16 | Yes |
| Chronic stress | Depression | TABU | NPN | 0.884 | 0.855 | 0.898 | 0.731 | 0.224 | 0.183 | 4 | No |
| Chronic stress | Anxiety | TABU | NPN | 1 | 0.887 | 0.993 | 0.878 | 0.993 | 0.878 | 16 | Yes |
| Age | MIND diet | TABU | NPN | 0.87 | 1 | 0.9 | 1 | 0.225 | 0.25 | 4 | No |
| Age | Blood pressure^§^ | TABU | NPN | 0.991 | 1 | 0.952 | 1 | 0.833 | 0.875 | 14 | Yes |
| Age | Chronic stress | TABU | NPN | 1 | 1 | 0.993 | 1 | 0.993 | 1 | 16 | Yes |
| Age | Marital status | TABU | NPN | 0.989 | 1 | 0.964 | 1 | 0.723 | 0.75 | 12 | Yes |
| Gender | Cognitive activity | TABU | NPN | 1 | 1 | 0.999 | 1 | 0.999 | 1 | 16 | Yes |
| Gender | MIND diet | TABU | NPN | 0.971 | 1 | 0.963 | 1 | 0.963 | 1 | 16 | Yes |
| Gender | Alcohol | TABU | NPN | 0.999 | 1 | 0.999 | 1 | 0.999 | 1 | 16 | Yes |
| Gender | Marital status | TABU | NPN | 1 | 1 | 1 | 1 | 1 | 1 | 16 | Yes |
| Education | Cognitive activity | TABU | NPN | 1 | 1 | 1 | 1 | 1 | 1 | 16 | Yes |
| Education | IRSAD decile | TABU | NPN | 0.88 | 1 | 0.965 | 1 | 0.905 | 0.938 | 15 | Yes |
| Social support | Physical activity | TABU | NPN | 0.993 | 0.688 | 0.963 | 0.711 | 0.903 | 0.666 | 15 | Yes |
| Depression | Social support | TABU | NPN | 0.999 | 0.841 | 0.984 | 0.838 | 0.984 | 0.838 | 16 | Yes |
| Anxiety | Depression | TABU | NPN | 1 | 0.872 | 0.997 | 0.785 | 0.997 | 0.785 | 16 | Yes |
| Resilient coping | Cognitive activity | TABU | NPN | 1 | 0.677 | 0.98 | 0.655 | 0.919 | 0.614 | 15 | Yes |
| Resilient coping | Perceived stress | TABU | NPN | 0.894 | 0.685 | 0.933 | 0.616 | 0.233 | 0.154 | 4 | No |
| Resilient coping | Chronic stress | TABU | NPN | 0.878 | 0.623 | 0.883 | 0.62 | 0.11 | 0.078 | 2 | No |
| Recent stressors | Perceived stress | TABU | NPN | 1 | 1 | 1 | 1 | 1 | 1 | 16 | Yes |
| Recent stressors | Chronic stress | TABU | NPN | 0.984 | 1 | 0.983 | 1 | 0.983 | 1 | 16 | Yes |
| Recent stressors | Marital status | TABU | NPN | 0.896 | 1 | 0.93 | 1 | 0.465 | 0.5 | 8 | No |
| Recent stressors | Depression | TABU | NPN | 0.902 | 1 | 0.876 | 1 | 0.274 | 0.313 | 5 | No |

*^*^ Arc coefficients for the given network. ^†^Arc coefficients averaged across subnetworks where they occurred. ^‡^Arc coefficients averaged across all potential subnetworks.* *^§^Attention to the management of that condition. INT = Inverse normal transformation, NPN = Non-paranormal transformation, Disc5 = Five-level discretization transformation, Disc3 = Three-level discretization transformation, TABU = TABU list algorithm, HC = Hill-climbing algorithm, MMHC = max-min hill-climbing algorithm, H2PC = Hybrid HPC algorithm. BMI = Body mass index, MIND = Mediterranean-DASH Intervention for Neurodegenerative Delay, IRSAD = Index of Relative Socio-Economic Advantage and Disadvantage.*
