# Supplementary material for: Psychosocial hierarchies of modifiable risk for Alzheimer’s disease: A networks analysis
Source: PLoS One. 2026 Mar 6;21(3):e0333148. doi: 10.1371/journal.pone.0333148 (PMC12965608; doi:10.1371/journal.pone.0333148)
Supplement: S1 File — Analyses evaluating the relationship between the regularized partial correlation network’s predictability statistic and other network metrics. (DOCX) [file pone.0333148.s006.docx]

## **S1 File. Assessing the regularized partial correlation network’s predictability statistic.**

An R^2^ statistic (predictability [1]) for each node was generated, using *mgm.fit*, as a reliable alternative to RPCN-associated centrality indices [2] (centrality [3]). Centrality indices and predictability represent the magnitude of per-node malleability, as influenced by its connections. For example, a node’s predictability score constitutes the maximum degree (as a percentage) to which its observed score is expected to change based on the combined variance of values across its connected nodes, as displayed by the RPCN. The interpretation of centralities is network-specific whereas predictability constitutes an absolute measure which may be generalised across datasets and networks [1]. Predictability was found to most strongly correlate with the strength centrality metric.

To support the interpretation of predictability within the present dataset, Pearson correlations (cor, method = pearson) tested the strength-predictability relationship. Additional correlations, between predictability and centrality indices of betweenness, closeness and expectedInfluence [1], were reported to provide context. A strong, and leading, correlation (*r* ≥0.70) with strength centrality was considered substantial evidence to support interpretation of predictability.

Analysis between predictability and CS-coefficients revealed a strong and significant correlation between predictability and strength, *r*(18) *=* 0.77, *P <*0.001. Correlation aligned with the uppermost findings of an earlier study [2], supporting the interpretation of predictability. Predictability and expectedInfluence were moderately and significantly correlated, *r*(18) *=* 0.64, *P* = 0.002, and weak, non-significant, correlations were found between predictability and respective measures of closeness*, r*(18) *=* 0.29, *P =* 0.22, and betweenness*, r*(18) *=* 0.29, *P* = 0.22.

## References

1. Haslbeck JMB, Fried EI. How predictable are symptoms in psychopathological networks? A reanalysis of 18 published datasets. 2017 [cited 6 Mar 2024]. doi:10.1017/S0033291717001258

2. Fried EI, Eidhof MB, Palic S, Costantini G, Huisman-van Dijk HM, Bockting CLH, et al. Replicability and Generalizability of Posttraumatic Stress Disorder (PTSD) Networks: A Cross-Cultural Multisite Study of PTSD Symptoms in Four Trauma Patient Samples. Clinical Psychological Science. 2018;6: 335–351. doi:10.1177/2167702617745092

3. Epskamp S, Borsboom D, Fried EI. Estimating psychological networks and their accuracy: A tutorial paper. Behav Res Methods. 2018;50: 195–212. doi:10.3758/s13428-017-0862-1
